# Genome Aging in Women: Hematopoietic Loss of X Chromosome as An Exemplar

**DOI:** 10.1111/acel.70778

**Published:** 2026-10-21

**Authors:** Xihan Guo, Xueqin Dai

**Affiliations:** ^1^ School of Life Sciences Yunnan Normal University Kunming Yunnan China; ^2^ Engineering Research Center for Valorization of Unique Bio‐Resources in Yunnan, Ministry of Education Yunnan Normal University Kunming Yunnan China; ^3^ School of Biomedical Engineering Kunming Medical University Kunming China

**Keywords:** aneuploidy, chromosome X linked competition, loss of X chromosome, somatic mosaicism, Turner syndrome, X chromosome inactivation, X chromosome reactivation

## Abstract

Genome aging refers to age‐related acquisition and expansion of *de novo* mutations in somatic cells, and is now increasingly viewed as a central driver of organismal aging. Loss of X chromosome (LOX), the state in which one X chromosome (ChrX) is missing, represents the most common genetically inferred type of acquired mutation in women. Recent technological advancements, along with the growing availability of large‐scale cohorts for association studies, have provided powerful new insights into this phenomenon. The frequency and clone size of LOX increase significantly with age: with over 10% of women aged 55 years or older showing detectable LOX in blood. LOX preferentially affects the inactivated ChrX derived from either parent, or in some cases, affects the ChrX from one specific parent. Besides aging, the acquisition and expansion of LOX are shaped by factors related to ChrX itself, genome‐wide inherited and acquired variants, and lifestyle and environmental stressors. Due to the critical biological roles of ChrX, even in its inactive state, LOX impairs cellular functions and is strongly linked to hematological malignancies and a range of benign diseases. Understanding the mechanisms driving the causes and consequences of LOX may expand the sex‐biased genomic architecture of aging and uncover strategies for disease prevention in women. In this review, by integrating the most recent progress in LOX studies with unique biology of ChrX and perspectives from aneuploidy, we provide a synthesized and updated understanding of the rise, expansion and consequences of blood LOX, while also posing key questions for future research.

## Graphical abstract

As studies at the intersection of genetics and aging are rapidly advancing, genome aging provides a novel mechanism by which aging phenotypes occur. In this Review, the authors examine the rise, expansion, and the consequences of X chromosome loss in female blood cells. They summarize the known clinical associations, outline emerging mechanistic insights, highlight remaining challenges, and suggest future directions.

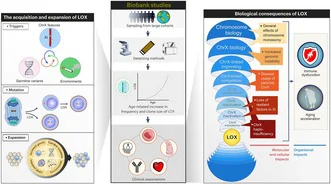

**Abbreviations**
AD: Alzheimer's disease
AML: acute myeloid leukemia
CF: cell fraction
CH: clonal hematopoiesis
CHIP: clonal hematopoiesis of indeterminate potential
ChrX: chromosome X
ChrY: chromosome Y
GWAS: genome‐wide association study
HSPCs: hematopoietic stem and progenitor cells
iPSCs: induced pluripotent stem cells
LOX: loss of X chromosome
LOY: loss of Y chromosome
mCAs: mosaic chromosomal alterations
MoChA: MOsaic Chromosomal Alterations
PARs: pseudoautosomal regions
PCD: premature centromere division
PLCO: Prostate, Lung, Colorectal, Ovarian Cancer Screening study
SNP: single‐nucleotide polymorphism
TS: Turner syndrome
UKB: UK Biobank
Xa: active X chromosome
XCI: X chromosome inactivation
XCU: X chromosome upregulation
Xi: inactive X chromosome
XLC: ChrX‐linked competition
Xm: maternal X chromosome
Xp: paternal X chromosome

## 1 Loss of X Chromosome: When Two are Too Many

Studies at the intersection of genetics and aging are rapidly advancing, revealing that age‐related accumulation and expansion of *de novo* mutations in somatic cells, a process termed genome aging (Figure 1A), are common in healthy, normal‐appearing tissues across organ systems (Ren et al. 2024; Brunner et al. 2026). A prime example is the hematopoietic system, in which hematopoietic stem and progenitor cells (HSPCs) contribute to the daily production of billions of blood cells via physiological hematopoiesis. Clonal hematopoiesis (CH) occurs when mutated HSPCs outcompete wildtype HSPCs and produce populations of leucocytes harboring the same mutations in persons with otherwise normal hematologic characteristics (Singh et al. 2026). The size of mutations ranges from small, single‐nucleotide variants to larger, chromosome‐level alterations (Dai and Guo 2021). Single‐nucleotide variants in genes associated with myeloid cancer in healthy individuals refer to CH of indeterminate potential (CHIP; Figure 1B) (Vlasschaert et al. 2024). Alterations encompassing ≥ 1–2 Mb refer to mosaic chromosomal alterations (mCAs; Figure 1B) (Dai and Guo 2021). Data from large‐scale cohorts have shown that mCAs are not evenly distributed across the genome. The most ubiquitous age‐related hematopoietic clones of mCAs involve the Y chromosome (ChrY) in men (46,XY) and the X chromosome (ChrX) in women (46,XX) (Dai and Guo 2021; Cheng et al. 2023).

**FIGURE 1:**
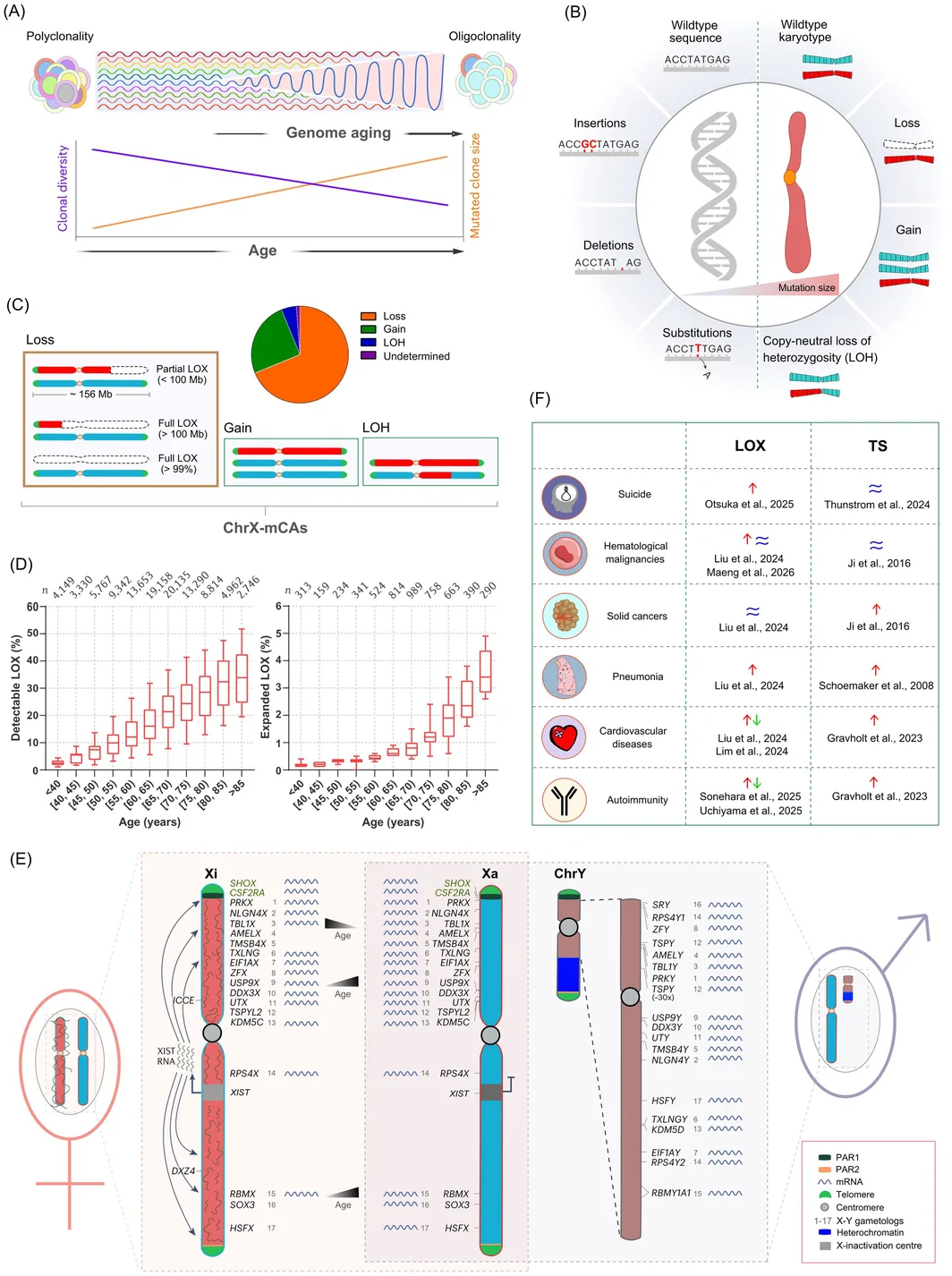
Loss of ChrX (LOX) is a prominent example of genome aging in women. (A) The definition of genome aging. From the time of fertilization onwards, our personal genome is continuously damaged by unavoidable endogenous and exogenous factors. Thus, each cell of an individual carries a uniquely modified version of the original genome in fertilized oocyte. In this sense, young and healthy individuals are comprised of diverse cell clones with unique genomes (polyclonality). Once a cell acquires mutations that can confer advantages over other cells, the homeostasis of diverse clones is disrupted, resulting in progressive evolution towards an oligoclonal population (oligoclonality). (B) Clonal hematopoiesis (CH) can be categorized based on the size of CH‐driven mutations, ranging from small, single‐nucleotide variants to larger, chromosome‐level alterations. Single‐nucleotide variants (including point mutation, small insertion, or deletion) in genes associated with myeloid cancer present at ≥ 2% variant allele fraction in healthy individuals refer to clonal‐hematopoiesis of indeterminate potential (CHIP). Mosaic chromosomal alterations (mCAs), defined as alterations encompassing ≥ 1–2 Mb, can be divided into three major subtypes, namely copy‐number loss and gain and copy‐neutral loss of heterozygosity (LOH). Setting the threshold to 1 Mb was due to our inability to distinguish gain, loss, and copy‐neutral LOH events < 1 Mb in size by MoChA, the commonly used mCAs calling software. (C) Illustration of the most commonly seen subtypes of ChrX‐mCAs. These include gain of ChrX and copy‐neutral LOH of ChrX and LOX, which could affect whole or fragments of ChrX (≥ 1 Mb). Whole chromosome ChrX‐mCAs typically result from mitotic chromosome missegregation, and segmental ChrX‐mCAs are the consequence of improper repair of DNA strand breakage. Depending on the size of the variant region, LOX can be sub‐classified into partial LOX and full LOX. Pie chart in the inset shows the fraction of each ChrX‐mCAs subtypes from the National Heart, Lung, and Blood Institute Trans‐Omics for Precision Medicine program, with 68.8% of ChrX‐mCAs being the LOX. Data was compiled from (Jakubek et al. 2023). (D) The prevalence of LOX (left panel) and expanded LOX (right panel) increases with age (centre line, median; box, interquartile range; whiskers, 1.5 × interquartile range). LOX occurs in a clonal subpopulation of blood cells and is detected more frequently in individuals above the age of 55 years. The prevalence of LOX is substantially reduced when restricted to CF > 5%. Of note, expanded LOX is detected more frequently in individuals above the age of 65 years. Data reanalyzed from (Liu et al. 2024). (E) Sex chromosome complements of female and male cells and a brief comparison of human ChrX and ChrY. All euploid somatic cells of women and men have 46 chromosomes, 45 of them are shared: 22 pairs of autosomes and one Xa. The 46th chromosome is Xi in females and ChrY in males. Although being largely non‐homologous, human ChrX and ChrY have two homologous pseudoautosomal regions (PARs) on their distal arms that regularly pair and cross over during meiosis of men. Outside PARs, human ChrX and ChrY express 17 pairs of homologous but non‐identical genes, referred to as X–Y gametologs, which have evolved separately owing to the absence of recombination between ChrX and ChrY during male meiosis, thus having divergent sequences and copy numbers to different degrees. Among them, 10 ChrX gametologs are constitutively escaped from ChrX inactivation (Owens et al. 2024). Interestingly, some escapees exhibit age‐related escaping potential (Rocca et al. 2026). Besides X–Y gametologs, some key genes or genetic elements involved in LOX are also annotated. (F) A brief comparison of disease spectrum associated with LOX individuals and Turner syndrome (TS). ↑, disease risk is increased; ↓, disease risk is decreased; ↑↓, the association has not been definitely established; ≈, neutral to disease risk. Of note, heterogeneity arising from LOX ascertainment methods and diseases clinical diagnoses, as well as variations in population structure across cohorts including participants' health state, geographical location and ancestry, may bias LOX studies, leading to conflicting results and affecting result interpretation.

Human ChrX spans about 156 million base pairs (Mb) and habits over 800 protein coding and 600 non‐coding genes (Ross et al. 2005). The finding that female cells tend to lost one of their two ChrX is not new. In 1963, Jacobs and colleagues uncovered that loss of ChrY (LOY) and loss of ChrX (LOX) occur frequently in blood cells of aging men and women, respectively (Jacobs et al. 1963). We are now in a watershed moment in LOX study. During the past decade, research efforts using sensitive sequencing technologies and advanced analytical methods, coupled with the growth of global biobanks in scale, have revolutionized our knowledge of LOX. In a landmark study, epidemiological data from 883,574 females across 8 biobanks have shown that LOX rises sharply with age and is a major risk factor in multiple age‐related diseases (Liu et al. 2024), therefore have attracted considerable interest in LOX. While these developments are transforming LOX from observational studies into a data‐rich field, several important questions also arising. For example, how does LOX become the most ubiquitous mCAs in females? Why ChrX tends to be lost? Which one of the two ChrX is preferentially affected by LOX? What role does the genetic predispositions and environmental exposure play in modulating LOX? Whether LOX contributes casually to aging and age‐related disease and if so, how and to what extent? Is LOX measured in blood a marker of shared mechanisms contributes to disease susceptibility or a direct contributor to disease development? Could LOX serve as an early biomarker of aging and disease risk? While these and other questions have widespread implications for women aging and health, they have been difficult to address in detail owing to a lack of empirical data.

This Review draws on insights gained from decades of lower‐resolution cytogenetic studies and more recent higher‐throughout omics and population‐based approaches, not only to provide a comprehensive summary of LOX, but also to illustrate fundamental principles of genome aging in women by taking LOX as an example. The organizational logic for this article begins with the definition of LOX and its prevalence and characteristics in blood of general female populations. We then illustrate the onset and clonal dominance of LOX in blood, with a specific focus on the effects of germline variants, environmental factors, ChrX features and cell competition. Importantly, we discuss the clinical associations of LOX by evaluating recent findings from large, population‐based cohort studies. To better understand the role and mechanism of LOX in disease development, it is helpful to be familiar with the biological consequences of LOX. We synthesize the biology of LOX around two interconnected themes. Firstly, we summarize the potential mechanisms through which LOX can have impacts on molecular and cellular function due to ChrX haploinsufficiency, loss of resilient factors in second ChrX, skewed usage of parental ChrX, increased genomic instability and other alterations from a general cellular response to chromosome loss. Secondly, we discuss organismal impacts of LOX, including impaired immunity and accelerated aging. Finally, we conclude with open questions of this field. In a broader sense, ChrX‐mCAs (Figure 1C) are discussed in parallel because LOX was analyzed together with other ChrX‐mCAs events in a subset of studies. LOX has been most well studied in human peripheral blood thus far. To enable an in‐depth discussion of LOX, we focus primarily on the hematopoietic system and provide only a brief overview of LOX in other tissues and cells (Box 1), and we recognize that the findings from the hematopoietic system may be able to extend to other self‐renewing tissues.

BOX 1 **LOX in tissues and cells beyond hematopoietic system.**

First discovered in blood cells in 1963, LOX has since been observed in various other cell types. It has long been known that human stem cells with XX chromosome complement derived from diploid parthenogenetic embryos are unstable and tend to lose entire ChrX or ChrX segments when cultured in vitro for long periods (Robertson et al. 1983). In agreement, LOX is frequently detected in XX mouse embryonic stem cells (Pasque et al. 2018; Stanton et al. 2026). In addition, cells of the hippocampus and prefrontal cortex in patients with AD have increased rates of ChrX aneuploidy (ChrX gain in 10%–20% of cells and loss in 80%–90% of cells) (Yurov et al. 2014). Genome sequencing data found that LOX is prevalent in the dorsolateral prefrontal cortex of females (Graham et al. 2019). Besides, the observation that overweight and obese women had an increased risk of blood LOX (Young et al. 2024) brings questions of whether LOX occurs in metabolic organs, and if so, whether LOX influences metabolic traits. Undoubtedly, these data are essential to elucidate broader impacts of LOX on human diseases. Beyond LOX, ChrX‐mCAs are 3.5‐times more frequently observed in buccal cells as compared with leukocytes (Machiela et al. 2016), suggesting ChrX‐mCAs have more selective advantages within buccal tissue than in blood when in competition with wildtype clones.

In addition, LOX occurs in patients with sex chromosome aneuploidy. The four common sex chromosome aneuploidy disorders are 45,X (TS), 47,XXX (triple X syndrome), 47,XXY (Klinefelter syndrome), and 47,XYY (Jacobs syndrome). LOX occurring during the post‐zygotic development may contribute to the birth of TS subjects. The major cause of TS is the mitotic loss of a ChrX or ChrY in a diploid 46,XX or 46,XY conception during the post‐zygotic development (Hook and Warburton 2014). Of note, TS subjects usually have variable karyotypes, characterized by complete or partial ChrX monosomy (Cameron‐Pimblett et al. 2017), suggesting LOX has occurred at different times during their post‐zygotic development. Subjects with extra ChrX are usually sub‐infertile or infertile because the extra ChrX impairs the production of germ cells (Lu et al. 2024). Theoretically, LOX provides a way to delete the extra ChrX during gametogenesis, thereby giving rise to the production of gametes with normal sex chromosomes. Indeed, although the peripheral blood lymphocytes of Klinefelter syndrome do not show signs of LOX (Kawakami et al. 2004), LOX has been observed in mature Sertoli cells, in which LOX allows focal spermatogenesis in Klinefelter syndrome (Winge et al. 2024). LOX has also been observed during the reprogramming of human 47,XXY fibroblasts or mouse 41,XXY fibroblasts to iPSCs (Hirota et al. 2017). Thus, LOX offers valuable insights into early intervention strategies for infertility in sex chromosome hyperploidy.

## 2 Defining LOX and Its Fundamental Characteristics

### 2.1 The Definition of LOX

As LOX has been increasingly studied, clarity of terminology is essential in this field. There were many ways to define LOX depending on research perspective. Initially, ChrX‐mCAs were defined as the large‐scale ChrX mosaicism (> 2 Mb) including the copy‐number loss and gain of ChrX and copy‐neutral loss of heterozygosity of ChrX (Machiela et al. 2016) (Figure 1C). Among 59 LOX events screened from 38,303 women, 47 of them (79.7%) affect a length > 150 Mb and the remaining affects a length 2–92 Mb. Compared to ChrX loss and gain, ChrX copy‐neutral events are much smaller in affected size and substantially varied in affected region (Machiela et al. 2016) (Figure 1C). Liu et al. (2024) defined LOX with a length ≥ 100 Mb as full LOX and these < 100 Mb as partial LOX and this criterion is followed by subsequent studies (Barnao et al. 2026). Full LOX is a major subtype of LOX, representing 98% of LOX events detected in the FinnGen cohort (Liu et al. 2024). Thus, partial LOX is excluded in most recent studies, and our discussion below therefore focuses on full LOX. With the advances in sensitive methodology (Box 2), LOX is readily detectable even in low fraction of leukocytes. In aiming to discover a more robust phenotypic association with LOX, some studies restricted the analysis to expanded LOX, with a threshold cell fraction (CF) over 5% or 10% (i.e., LOX present in at least 5% or 10% of peripheral leukocyte DNA) (Zekavat et al. 2021; Liu et al. 2024; Sonehara et al. 2025). Considering this, the term mosaic LOX denotes that LOX occurs in certain fractions of cells in whole organ or whole organism.

BOX 2 **Methodologies for LOX detection.**

Several methods, with different levels of sensitivity, advantages and disadvantages, have developed to detect LOX.

- Cytogenetics

The metaphase karyotyping, coupled with chromosome banding, is the earliest cytogenetic approach used to detect LOX (Jacobs et al. 1963). However, as this method needs actively dividing cells (such as lymphocyte cultures) to prepare sufficient metaphase chromosome images, it has limited utility in many non‐dividing somatic tissues. In addition, although in vitro culturing itself does not increase the predisposition of LOX (Catalán, Surrallés, et al. 2000), it would introduce additional mutations and selection artifacts. To circumvent these limitations, subsequently developed FISH could detect LOX in interphase nuclei. Using fluorescently labeled probes that hybridize to centromeric regions of ChrX, FISH enables the direct visualization of ChrX copies in single cells (Invernizzi et al. 2004). The unique characteristics of Xi, such as hyper‐condensation and depletion of acetylated histones, enable us to identify which ChrX is lost in interphase cells. For example, by combining immunolabeling of acetylated histone H4 with specific antisera and FISH with a ChrX centromere‐specific DNA probe, cells with loss of Xi may be identified by their lack of histone H4 acetylation (Jeppesen and Turner 1993). Moreover, the late‐replicating feature can be used as an identifier of Xi during mitosis via labeling with 5‐bromo‐2′‐deoxyuridine (Catalán, Falck, and Norppa 2000).

- Polymerase chain reaction (PCR)‐based techniques

By using specific primer pairs representing several different regions of ChrX, quantitative PCR‐based techniques provide an alternative method to measure LOX (Miozzo et al. 2007; Machiela et al. 2016). Compared to cytogenetic approaches, quantitative PCR‐based technologies are less labor‐intensive and require less technical expertise. More importantly, if parental samples of LOX individuals are available, the parental‐of‐origin of the retained ChrX can be determined by amplifying the polymorphic markers located on the distal short arm of ChrX via PCR (Skuse et al. 1997; Martínez‐Pasarell et al. 1999). The droplet digital PCR provides a more sensitive approach to absolutely quantify ChrX copy number and accurately estimate the degree of LOX mosaicism (Kikuchi et al. 2026). Notably, a specific technique, named the SinChro assay, has been designed to measure low‐level LOX at the single‐cell solution by employing the multiplexed single‐cell droplet PCR (Tsuchiya et al. 2024). However, an important caveat of quantitative PCR‐based techniques is that full and partial LOX could not be separated.

- Microarray‐based techniques and DNA sequencing

A true game changer is the single‐nucleotide polymorphism (SNP) array, which was initially engaged for diagnosing the precise karyotype of TS (Prakash et al. 2014). Subsequently, it has been widely used to detect LOX at the population level, particularly in biobank‐scale studies (Liu et al. 2024). By measuring intensity values across 82,387 probes in all regions excluding two PARs, SNP array‐based techniques allow reliable inference of copy number variation across the ChrX, thereby providing a superior resolution in comparison to cytogenetics and PCR‐based analyses (Graham et al. 2019). The SNP array‐based techniques have many advantages in detecting LOX, including high throughput and sensitivity and different cell types can be analyzed (Biesecker and Spinner 2013). However, array‐derived intensity signals capture only a subset of predefined loci across ChrX. To provide a ChrX‐wide coverage, whole‐exome sequencing (Liu et al. 2024; Berghaus et al. 2025) or whole‐genome sequencing (Jakubek et al. 2023) has recently been employed to measure LOX. To call LOX from SNP array and sequencing data, the MOsaic CHromosomal Alterations (MoChA) software has been widely used (Loh et al. 2018). Overall, microarray‐based techniques and DNA sequencing not only enable a more accurate estimation of ChrX copy number, but also help to determine the genome‐wide germline predispositions to LOX and other CH events that co‐occurred with LOX by integrating the germline variant and CH data.

- RNA sequencing

RNA sequencing data are also being used to examine LOX. Deep analyzing the expression signatures of selected ChrX genes in transcriptomic data can lend insight into functional LOX (Qi et al. 2023; Stanton et al. 2026). Since the relative ChrX read depth correlates well with ChrX‐wide expression in dorsolateral prefrontal cortex of female brain (Graham et al. 2019), functional LOX may be considered a proxy for LOX in females. A recently developed method called allele‐specific single‐cell RNA seq is able to split the ChrX‐derived transcripts by allele of parental origin (Talon et al. 2021; Allsop et al. 2025), thereby being able to provide a potential parental‐of‐origin resolution to functional LOX. Despite these advantages, single‐cell sequencing requires significant costs and efforts to address artifacts risen from in vitro whole‐genome amplification (Coorens et al. 2025).

Overall, this expanded toolbox of microcopy, molecular biology, and computational assays has fueled, and will continue to fuel, exciting discoveries in our understanding of LOX. Considering each technology has strengths and weaknesses, a combination of different methods can achieve comprehensive discovery and accurate analysis. Moreover, each method may introduce artificial noise due to the technical limitations. For example, it is plausible that a low fraction of Xi has been removed together with nuclear debris during DNA purification because Xi preferentially locates to nuclear peripheral. If so, it may partially explain why LOX preferentially affects Xi and why only expanded LOX sometimes is associated with phenotypes. In this sense, in situ approaches such as FISH remain important valid diagnostic tools for DNA‐based LOX data.

Aneuploidy refers to a genetic state that the copy number of chromosomes or chromosome arms in a cell has either amplified (trisomy) or deleted (monosomy) (Fusari and Milán 2026). While LOX and ChrX monosomy are similar and interchangeable concepts, as we have presented in this Review, it is important to note that they are not synonymous: whereas LOX refers to an elevated rate of ChrX losing, ChrX monosomy describes the state of having only one copy of ChrX. In other words, LOX is the cellular process that leads to ChrX deletion, and ChrX monosomy is one genetic result. In principle, there are two ways for human cells to become ChrX monosomy: LOX in females and LOY in males.

Of note, male cells (46,XY) are also likely to lose their sole ChrX, but its prevalence is context‐dependent. While early cytogenetic evidences have shown that LOX also occurs in cultured lymphocytes of men (Song et al. 2009; Nuebling et al. 2021), even with a frequency comparable to women (Catalán, Surrallés, et al. 2000), however, ChrX genotyping array intensity data from 196,219 men in UK Biobank (UKB) found no evidence for LOX in blood DNA (Zhou et al. 2021). This has been verified in a small male cohort through the multiplexed single‐cell droplet PCR (Tsuchiya et al. 2024). These findings suggest that LOX occurs frequently in male cells in vitro, where there is less negative selection against them; however, these cells rarely expand in vivo, reflecting that a strong selective pressure is exerted to maintain ChrX ploidy in males. Therefore, unless otherwise indicated, LOX discussed in the following sections refers to the full LOX restricted in females.

### 2.2 The Prevalence of LOX Increases With Age

It is widely known that LOX is an age‐related acquired somatic mutation. In ex vivo lymphocytes from 655 females aged from birth to 80 years, the rate of LOX determined by cytogenetic approaches is highly correlated with age (Russell et al. 2006). Liu et al. (2024) have found that the prevalence of detectable LOX and expanded LOX (CF > 5%) increases continuously with age (Figure 1D). Specifically, this study includes 8 biobanks of 883,574 female participants from Europe and East Asia, with 105,286 LOX carriers (11.9%) and 5475 expanded‐LOX carriers (0.6%). More recently, LOX is detected in 14,322 of 184,053 females (17%) from two large Danish cohorts, with LOX prevalence increasing with age (Maeng et al. 2026). Similarly, fraction of subjects with ChrX‐mCAs increases continuously with age (Cheng et al. 2023; Jakubek et al. 2023). While the prevalence of LOX consistently increases with age across cohorts, it varies substantially in different cohorts, even these from same countries. For instance, fraction of LOX females is less than 20% in women aged 60–70 years from the Copenhagen hospital biobank, but is as high as over 35% in women of the same age group from the Danish Blood Donor Study (Maeng et al. 2026).

Interestingly, the prevalence of LOX in Mass General Brigham Biobank increases linearly from 3.5% in women aged < 40 years to 33.5% in women aged 80–85 years old, after which it drops to 26.3% in women aged ≥ 85 years (Liu et al. 2024). These data suggest that LOX burden may not increase infinitely with age and have an upper limit, probably because the CF of LOX above a threshold becomes incompatible with life. Considering this, the longitudinal dynamic of LOX in centenarians is an interesting research theme because centenarians represent a population of successful aging. In this landmark population‐based cross‐biobank study from Liu et al. (2024), centenarians are rare and generally included in the age bin of aged ≥ 85 years, hurdling our understanding of LOX in centenarians. In an earlier study (Bukvic et al. 2001), lymphocytes isolated from three young subjects (20–22 years old) and 11 centenarians or near‐centenarians (98–102 years old) were subjected to LOX detection by fluorescence in situ hybridization (FISH) and the results showed the frequency of LOX in young women was ~1.7% but was ∼22% in centenarian females. Overall, recognition of age‐related LOX represents a window on the process of genome aging in women.

### 2.3 Skewed LOX: Which ChrX Is Preferentially Affected?

Human sexes are distinguished by differences in sex chromosome dosage, that is women having two ChrX (i.e., bi‐allelic) and men having one ChrX (i.e., hemizygous) and a ChrY (Figure 1E). Although largely non‐homologous, human ChrX and ChrY are homologous in two tiny regions that regularly pair and cross over during meiosis of men, and therefore are termed as pseudoautosomal regions (PARs). Outside PARs, human ChrX and ChrY express seventeen pairs of homologous but non‐identical genes, referred to as the gametologs (Owens et al. 2024). Gametologs have global roles in modulating autosomal gene expression, even small differences in their function and regulation may shape phenotypic differences between male and females (DeCasien et al. 2025).

The two ChrX in female cells differ not only in their parental origins, but also in their (epi)genetic variations. In most mammals, to equalize the expression of ChrX genes between females and males, one ChrX of females is randomly chosen to be transcriptionally inactivated (Loda et al. 2022). This process, termed as ChrX inactivation (XCI), silences all but one ChrX in diploid cells of both sexes. XCI is initiated by *cis* expressed long non‐coding RNA XIST in early embryonic stem‐cell differentiation. In addition, Xi exhibit hypermethylation at gene promoters and hypomethylation at gene bodies, suggesting XCI is also coordinated by XIST‐independent strategies (Hellman and Chess 2007). Hence, XCI results in two epigenetic states of ChrX, the active (Xa) and inactive (Xi) (Figure 1E). The Xi, also known as Barr body, adopts a condensed three‐dimensional (3D) structure and generally locates at nuclear periphery (Jégu et al. 2017; Loda et al. 2022). XCI pattern is stable, with the same parental‐of‐origin ChrX maintained as Xi in subsequent cell divisions. Females are therefore genetically mosaic because random XCI creates two heterogeneous clones of cells expressing either their maternal ChrX (Xm) or paternal ChrX (Xp) (Migeon 2006). Notably, mosaic expression of ChrX in females may lead to function variation if two ChrX alleles are polymorphic (Migeon 2006). Beside XCI, ChrX upregulation (XCU) increases Xa expression in both sexes, thereby balancing gene expression relative to autosomes that are present in two copies (Deng et al. 2014). Like XCI, XCU is also initiated in early embryonic development with dynamic and stabilized patterns in adult tissues (Li and Duan 2022).

Human XCI is incomplete, with up to 23% of the total ~1098 Xi genes able to escape from XCI (Ross et al. 2005; Tukiainen et al. 2017). A portion of escaping genes (termed escapees) are classified as constitutive because they remain active in most cell types and developmental stages; in contrast, other escapees are facultative as they are activated in a cell‐type, developmental stage, or age‐specific way (Loda et al. 2022). XIST/Xist RNA is recently found to silence escapees well beyond early embryogenesis (Hauth et al. 2026). In addition, deviations from the expected random XCI, known as XCI skewing, are common in some tissues of the general female population (Shvetsova et al. 2019) and increase substantially in aging females (Busque et al. 1996). XCI skewing, which creates a circumstance in which females have either Xm or Xp being active in over 75% of their cells, is tissue‐dependent, with a higher proportion in blood‐derived tissues (Roberts et al. 2022).

Since female cells have two non‐identical ChrX, is there a preferential choice of which ChrX to be lost? That two ChrX of females have different susceptibility to loss has been indicated by studies of patients with Turner syndrome (TS), a disorder of sex chromosome aneuploidy in females characterized by complete or partial ChrX monosomy (Cameron‐Pimblett et al. 2017). One major cause of TS is mitotic loss of a ChrX or ChrY in a corresponding 46,XmXp or 46,XmY conception during the post‐zygotic development (Hook and Warburton 2014). Depending on the parental origin of ChrX, TS can be divided into 45,Xm and 45,Xp. While the predicted ratio of 45,Xm and 45,Xp should be 1:1, the observed ratio, however, is over 3:1 (Jacobs et al. 1997). One possible explanation is preferential loss of Xp or ChrY in early pregnancies. In agreement, somatic LOX in female blood cells preferentially affects one specific ChrX: Based on LOX profiles of UKB females, mathematical and computational modeling has revealed that two distinct LOX events occurring at rates varying by a factor of 140, reflecting that losing one ChrX is more plausible than losing the other one (Watson and Blundell 2023). Such bias could be explained by three nonexclusive models.

Firstly, in case of LOX, the ChrX homolog destined to be lost is theoretically much more likely the Xi, because retaining of Xa maintains an adequate ChrX gene dosage for cell survival. Early studies proposed that Xi, either derived from Xp or Xm, is preferentially lost during aging (Figure 2A) (Abruzzo et al. 1985). Moreover, methylation array analyses revealed that both LOX and gain of ChrX preferentially impact Xi (Machiela et al. 2016). Data from GTEx (genotype‐tissue expression) have shown that a majority of healthy female blood samples display low *XIST* expression (159/316, 50.3%) (Sadagopan et al. 2022). The strong association between LOX and decreased *XIST* expression (Qi et al. 2023) raises an intriguing possibility that Xi has been lost in these female blood samples. More recently, data from single‐cell analyses did suggest that Xi is always affected by LOX (Si et al. 2026).

**FIGURE 2:**
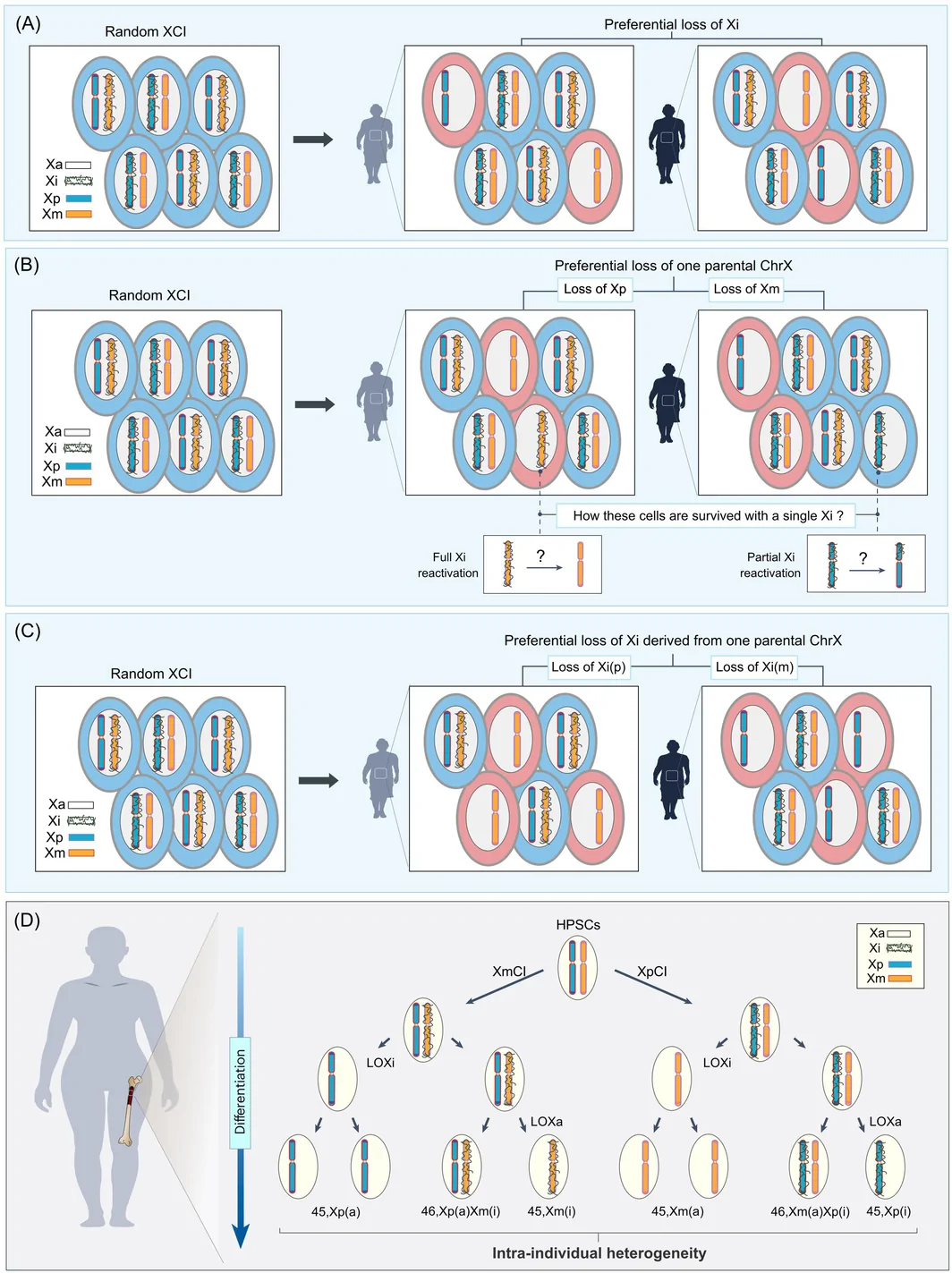
Non‐random choice of ChrX for somatic loss in females. Three models could be applied to account for the observed heterogeneity of loss of ChrX (LOX). (A) LOX preferentially affects the inactivated ChrX (Xi), either derived from the paternal ChrX (Xp) or maternal ChrX (Xm). (B) LOX preferentially affects one parentally derived ChrX, either in the form of active (Xa) or inactive (Xi) status. (C) LOX preferentially affects the Xi derived from either Xp or Xm. These models illustrate that the choice of ChrX to be lost is non‐random, thereby avoiding the lethal effect of losing both ChrX in female cells. Notably, these models are not mutually exclusive and may be operating in the same individual, leading to intra‐ and inter‐individual variations across tissues. (D) Detailed depiction of the LOX pattern in the hematopoietic system of a LOX carrier. According to the patterns of LOX mutation, the hematopoietic system of LOX individuals is a complex mosaic of genetically distinct clones, consisting of 45,Xm(a), 45,Xp(a), 45,Xm(i), 45,Xp(i), 46,Xm(a)Xp(i), and 46,Xp(a)Xm(i) cells, with the fraction of each clone being varied across individuals. 45,Xm(a), LOX cells expressing the Xm; 45,Xm(i), LOX cells retaining the inactivated Xm; 45,Xp(a), LOX cells expressing the Xp; 45,Xp(i), LOX cells retaining the inactivated Xp; 46,Xm(a)Xp(i); 46,Xp(a)Xm(i), normal cells expressing Xp but silencing Xm; ChrX, X chromosome; HSPCs, hematopoietic stem and progenitor cells; LOXa, loss of Xa; LOXi, loss of Xi; normal cells expressing Xm but silencing Xp; Xa, activated ChrX; Xi, inactivated ChrX; Xm, maternal ChrX; XmCI, inactivating the Xm; Xp, paternal ChrX; XpCI, inactivating the Xp.

Secondly, another model (Miozzo et al. 2007) postulated that one parent‐of‐origin ChrX, either in the form of Xa or Xi, is preferentially impacted by LOX (Figure 2B). Advances in genomic technologies and analytical approaches enable a deeper investigation into this model. Liu et al. (2024) employed allelic shift analysis in retained ChrX of LOX cases and revealed that LOX strongly favors one ChrX over the other, depending on 44 leading ChrX‐linked variants. A ChrX‐specific score on the basis of 44 independent variants could correctly predict which ChrX is likely to be retained in LOX cells (Liu et al. 2024). In addition, loss of Xa has been observed in certain contexts beyond the hematopoietic system. For example, functional LOX is detected in 25% (22/88) female mouse embryonic stem cell lines where both ChrX are transcriptionally active (Stanton et al. 2026), suggesting Xa is also frequently affected by LOX if mammalian cells have two Xa. Given that loss of Xa results in retaining the largely silent Xi, this raises the question of whether the remaining Xi will become partially or even fully reactivated upon loss of Xa (Figure 2B). This question is of particular interest because Xi tends to become partially re‐activated upon aging (see below) and blood cells are more susceptible to Xi reactivation than other cell lineages (Wang et al. 2016).

Finally, building on these two models, an attractive model is that LOX preferentially affects Xi derived from one parental ChrX (Figure 2C). Although speculative, this model represents an exciting prospect of future studies. Since DNA sequencing does not distinguish between Xa and Xi and parental DNA is unavailable for most biobank participants, testing this hypothesis is challenging. One promising approach is studying the landscape of LOX in women with completely skewed XCI (Gylemo et al. 2025) or a mouse model with one parent‐of‐origin ChrX being constitutively inactivated (Abdulai‐Saiku et al. 2025), because they provide a powerful system lacking the confounding effect of XCI mosaicism. As both XCI skewing and LOX are age‐related and preferentially affect one specific ChrX, it is therefore difficult to avoid the question of whether they could be related to each other. To address this gap, cohort‐based LOX studies in the future should ideally incorporate the knowledge on which parental ChrX is inactivated and which parental ChrX is lost.

## 3 The Acquisition and Clonal Expansion of LOX

Generally, the prevalence of aneuploidy may reflect frequent chromosome mis‐segregation (mutational bias), or fitness advantages associated with aneuploidy (selective bias) (Shih et al. 2023). Mechanisms by which LOX rise and expand are still being elucidated, but the mutation‐selection diagram is increasingly appearing. For instance, the observed high rates of ChrX‐mCAs (particularly LOX) but at overall lower CF suggests that clones with chrX‐mCAs may arise due to relatively weak positive selection of LOX but possibly high rates of ChrX missegregation during cell divisions (Jakubek et al. 2023; Si et al. 2026). This view is supported by a longitudinal characterization of LOX clonal dynamics in females of Prostate, Lung, Colorectal, Ovarian Cancer Screening study (PLCO) (Kelly et al. 2026). In the following subsections, we discuss molecular underpinnings that result in increased prevalence of LOX with age.

### 3.1 LOX Is Determined by the Genetic and Environmental Factors

Large‐scale population‐level genotypic data has been leveraged to identify statistically significant loci for LOX. An early genome‐wide association study (GWAS) in UKB has revealed four germline variants, each with tiny effects being associated with increased risk of LOX in *cis* (*DXZ1* and *DXZ4* located on ChrX) and in *trans* (*SP140L* and *HLA* located on autosomes) (Loh et al. 2018). A subsequent GWAS in US cohorts has found three genome‐wide significant loci associated with increased (*HLA‐B*) or decreased (*SP140L* and rs58502248) risk of LOX (Jakubek et al. 2023). The largest GWAS in 105,286 LOX carriers from Europe and East Asia has identified 56 common germline variants (minor allele frequency > 0.1%) at 42 independent loci that reached significance, with 34 variants associated with increased LOX risk and 22 variants associated with decreased LOX risk (Liu et al. 2024). In this sense, the combination of predisposition and protective variants dictates the overall risk of LOX within each personal genome. These variants have surprisingly weak overall effect sizes and are non‐randomly distributed in the genome, with most of them locating on chromosomes 6, 2, and X (Figure 3A). Among these genes, *TP53*, which encodes p53, is highly interesting, because experimental data have shown that RPE1 cells with LOX survived only when *TP53* was knocked out or knocked down (Chunduri et al. 2021). Moreover, research efforts in exploring less frequent variants have identified that one single rare missense variant (< 0.1% population frequency) in *FBXO10* confers a 2.1‐fold increased risk of LOX (Liu et al. 2024). A rare germline variant around *OR4C16* is associated with ChrX‐mCAs (Jakubek et al. 2023). These data suggest that variants of recent origin disproportionately contribute to LOX due to their large effect sizes (Lupski et al. 2011).

**FIGURE 3:**
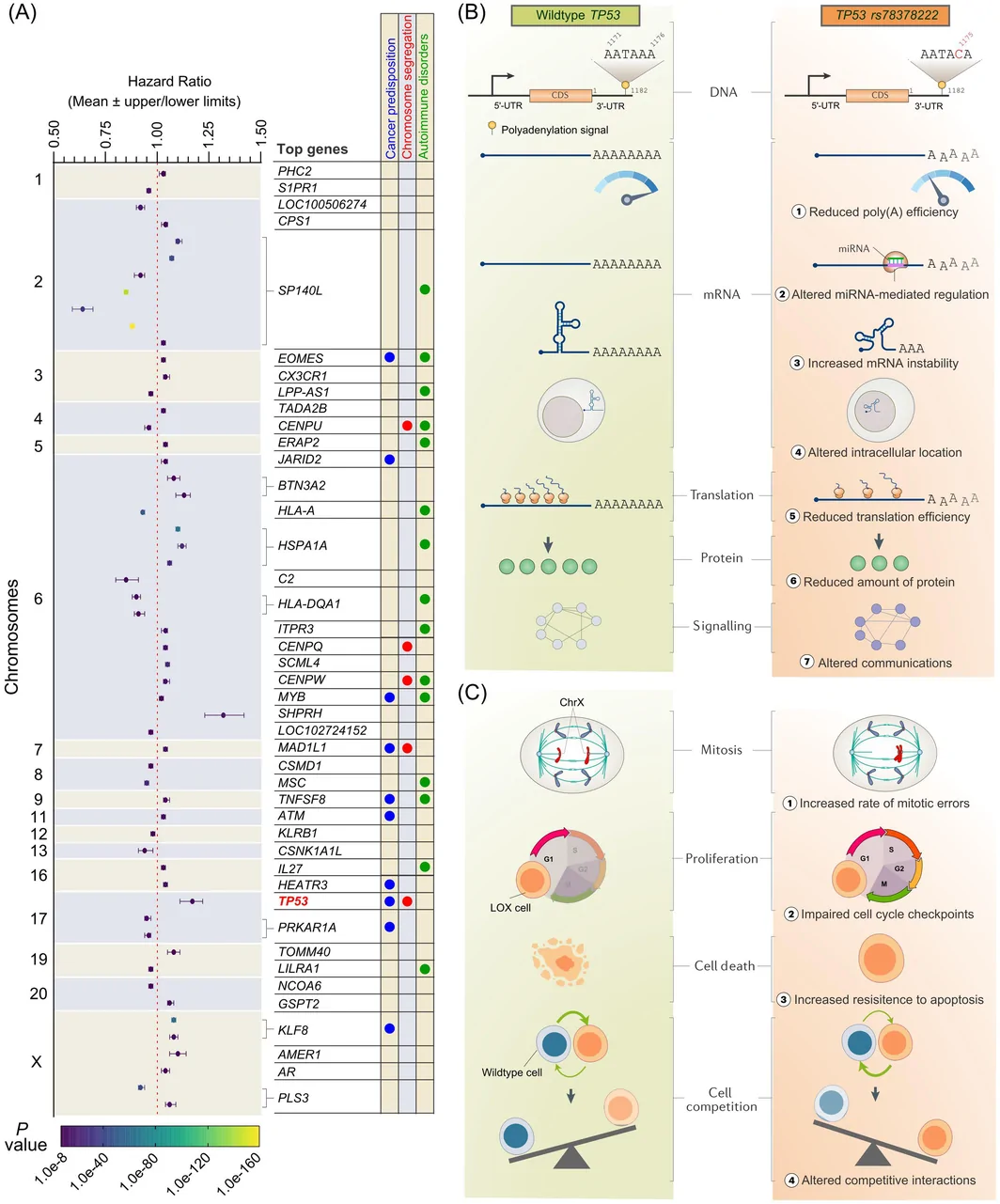
Overview of common germline variants associated with loss of ChrX (LOX). (A) 56 inherited genetic variants influence LOX predisposition. Particularly, common germline variants (the minor allele frequency > 0.1%) located within (*in cis*) and outside ChrX (*in trans*) have an important role in decreasing or increasing the risk of LOX. In general, the effect size of these variants is relatively small, suggesting LOX may result from the combined effects of these modest‐impact genes, rather than from the individual effects of high‐impact genes. Here these 56 variants are ranked according to their location on each chromosome, showing that they are not uniformly distributed in the genome. Genes involved in cancer predisposition (blue pot), chromosome segregation (red pot) and autoimmune disorders (green pot) are highlighted in the right panel. Data are compiled from (Liu et al. 2024). (B) Molecular consequences of *TP53* rs78378222. rs78378222 causes the base A at the position 1175 (counted from first nucleotide of the *TP53* 3′‐untranslated region (UTR)) to be substituted by C. Such substitution substantially reduces the efficiency of poly(A) formation (①) and increases the level of miRNA‐mediated repression (②). These events increase instability (③), alter intracellular location (④), and reduce translation efficiency (⑤) of TP53 mRNA, which subsequently reduces p53 protein (⑥) and alters p53‐mediated signaling networks (⑦). CDS, coding sequence. (C) Hypothesized routes to explore how rs78378222 increases risk of LOX. These four routes are increasing the rate of ChrX missegregation (①), allowing LOX to continue to cycle (failing in arresting LOX cells) (②), rendering LOX cells resistant to apoptosis (③), and increasing the competitiveness of LOX cells towards wildtype cells (biased competition) (④).

Several environmental factors have been identified to associate with LOX, though effect sizes are somewhat dependent on demographic characteristics of a cohort. A cross‐biobank study showed that ever‐smokers have an increased risk of expanded LOX (CF > 5%) (Liu et al. 2024), suggesting LOX expansion is associated with smoking. Longitudinal data from the PLCO cohort, however, revealed that smoking does not significantly impact the expansion of LOX (Kelly et al. 2026). In participants of the Danish Blood Donor Study, current smoking is mildly associated with LOX (Maeng et al. 2026). Moreover, results from UKB are mixed: smokers either do not have (Young et al. 2024) or have a slightly higher rate of LOX (Levin et al. 2022), probably due to methodological differences. Similarly, there is mixed evidence regarding the association between smoking and ChrX‐mCAs, either being no relationship (Machiela et al. 2016) or positively correlated (Cheng et al. 2023). While the rate of ChrX‐mCAs is significantly higher in smokers than non‐smokers, current smokers do not have more ChrX‐mCAs than ex‐smokers (Cheng et al. 2023), suggesting ChrX‐mCAs are irreversible when the smoking‐derived selection pressure is removed. Overall, evidence is lacking to demonstrate conclusively that smoking could drive LOX. Apart from smoking, females in UKB with moderate/high alcohol consumption had increased LOX risk, and moderate physical activity was negatively associated with LOX (Young et al. 2024). These data suggest LOX is an adaptive mutation that allows adaptation to environmental stresses by promoting clonal fitness (see below). While environmental risk factors of LOX are still under investigation, this field has been uneven, with more attention has paid to lifestyles and less to exogenous environmental exposures. In addition, although environmental factors are often studied independently from germline variants in the context of LOX, the environmental influence is unlikely to be free of any genetic influence.

Given that LOX is under both the genetic and environmental control, it is unsurprising to see that the prevalence of LOX or ChrX‐mCAs in most cohorts is biased by racial and ethnic groups (Liu et al. 2024). Jakubek et al. (2023) found that the fraction of each ChrX‐mCAs subtype in the affected population varies by genetic ancestry. Specifically, ChrX‐mCAs rate is higher in African or Hispanic ancestry but is lower in East Asian ancestry compared with European ancestry. By testing the association between ChrX‐mCAs and the estimated proportion of African ancestry on ChrX, the authors found a lower rate of ChrX‐mCAs in individuals with less than 25% of their ChrX derived from African ancestry compared with those with over 25% of their ChrX derived from African ancestry. Among ChrX‐mCAs subtypes, LOX is higher in rate in African ancestry and in Hispanic ancestry compared with European ancestry. East Asian females exhibit a lower rate of LOX than European and African ancestry groups, but a higher rate of ChrX gain than Europeans. The authors found the frequency of rs58502248, a variant located on chromosome 7 that associates with a decrease in risk of LOX by 41%, is 30 times more frequent in African ancestry (6.06%) than in European ancestry (0.02%), and this variant is totally absent in East Asia. The frequencies of the other two associated variants, however, do not show substantial divergence across ancestry. The inverse trend in the prevalence of LOX and frequency of variant rs58502248 across ancestry suggests environments/lifestyles or their interaction with germline variants may have major roles in the ancestry‐related bias of LOX. Although most genetic loci (52/56) associated with LOX are shared between participants with European and East Asia ancestry (Liu et al. 2024), whether the allele frequencies of these loci are differentiated between European and East Asia ancestry groups remains elusive. By contrast, the association between ChrX‐mCAs and ancestry (European, African and East Asian) is not observed in 38,303 women across multiple cohorts (Machiela et al. 2016). In agreement, no significant difference in the rate of ChrX‐mCAs was found between blacks and whites in a US cohort (Cheng et al. 2023). Considering these mixed results, more cross‐ancestry comparisons in large cohorts are needed to identify ancestry‐specific genetic and environmental determinants of LOX.

Together, germline variants and environmental factors, along with their tractable interaction effects, are major determinants of LOX mutation (Figure 4A). One challenge for the near future is to determine which variants or environmental factors are causally involved in LOX and to elucidate the underlying mechanisms.

**FIGURE 4:**
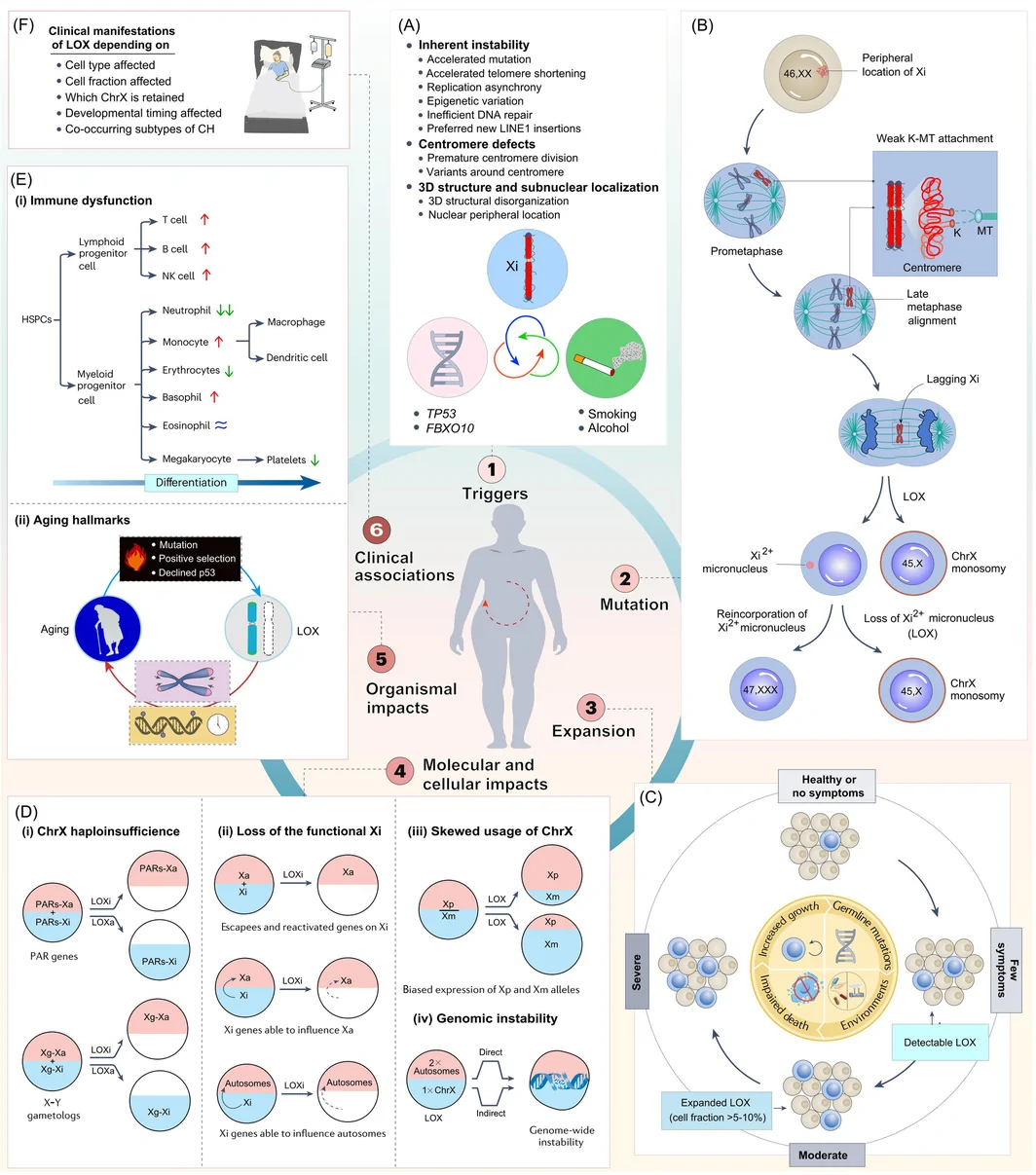
Schematic overview of the causes and consequences of loss of ChrX (LOX). (A) Triggers of LOX mutation. It is likely that factors lie within ChrX are primary triggers for LOX mutation. Factors not attributable to ChrX also play a role in LOX, such as germline mutations and environmental stressors, despite the mechanisms still being defined. Thus, intricate three‐way interplay among germline variants, environmental factors, and ChrX could trigger LOX mutation. (B) The micronucleation model of LOX mutation. Inactivated ChrX (Xi) has a much‐increased propensity for mitotic errors than activated ChrX (Xa). Compared to Xa, Xi is preferentially prone to premature centromere division and the following nondisjunction. Xi might degenerate more rapidly and is more susceptible to instability with age than Xa. Moreover, Xi tends to locate at peripheral regions of the nucleus at interphase. Thus, Xi might take longer than central‐located Xa to align at metaphase, thereby increasing the risk of unalignment. In addition, Xi centromeres are intrinsically defective, resulting in weak or incorrect kinetochore (K)‐microtubule (MT) attachments. All these features lead Xi prone to be incorrectly attached and/or nondisjunctioned during mitosis and to be incorporated into isolated micronuclei after mitotic exit. Thus, the karyotypes of daughter nuclei could be 45,X and 47,XXX (with two Xi engulfed in micronuclei). On one hand, micronuclei can be degraded by autophagy or, in some cases, be extruded, resulting in 45,X cell. On the other hand, micronuclei can be re‐incorporated into the nucleus, resulting in a 47,XXX cell with an extra Xi. Therefore, this model explains why both LOX and gain of ChrX preferentially affect Xi. Of note, loss of Xa may also occur occasionally via this model. (C) Clonal expansion of LOX cells. Under certain conditions, such as carrying specific genetic variants and exposing to environmental stressors, LOX confers a selective advantage by gaining proliferative advantage and/or increasing rate of survival (i.e., enhanced proliferation rate and/or heightened resistance to apoptosis). As a consequence, progenies of LOX cells will expand over time such that expanded LOX clone emerges. The threshold used to define expanded LOX usually falls within the range of 5%–10% of CF, depending on different studies and cohorts (Liu et al. 2024; Sonehara et al. 2025). Generally, LOX is biological and clinically related only when its clone size exceeds the threshold. (D) Molecular and cellular impacts of LOX. Four biological processes may contribute to the functional consequences of LOX. (i): Loss of Xa or Xi (LOXa or LOXi) gradually leads to haploinsufficiency in genes of pseudoautosomal regions (PARs) and ChrX gametologs (Xg). In response to ChrX haploinsufficiency, the remaining alleles will be upregulated to a certain extent. (ii): LOX preferentially affects Xi. LOXi depletes the expression of Xi‐linked escapees and genes that would be reactivated. In addition, loss of Xi leads to genome‐wide disturbance in gene expression because Xi can modulate autosome and Xa. (iii): LOX results in skewed usage of parental ChrX. Considering the functional difference between Xp and Xm derived from their sequence variation and epigenetic variation, skewed representation of them in ChrX monosomic cells has a non‐neglected impact on phenotypes. (iv): Functional consequences of LOX may also be impacted by co‐occurring genomic instability (such as autosomal mCAs and CHIP), either directly induced by LOX or indirectly co‐occurred with LOX by chance. Illustration design rationale was inspired by (Arnold 2022). (E) Organismal impacts of LOX. (i): Blood LOX is significantly associated with increased (↑) or decreased (↓) counts of specific blood cell types, suggesting LOX individuals may associate with immune dysfunction. The counts of some cell types remain unchanged (≈) in LOX individuals. (ii): The bidirectional causality between aging and LOX. On one hand, aging promotes the acquisition and clonal expansion of LOX via, e.g., inflammaging and declined p53 function. On the other hand, LOX may drive biological aging via accelerating telomere shortening and epigenetic clock. (F) Clinical associations of LOX. Pathogenic effects of LOX are heterogeneous due to a variety of contributing factors. CH, clonal hematopoiesis. Having proposed this model describing the cause and consequence of LOX, it is important to stress that, for the moment, many mechanisms of this model are not fully understood.

### 3.2 ChrX, Particularly Xi, Is Susceptible to Missegregation During Mitosis

It is known that there is an enhanced mutation rate on ChrX relative to autosomes in both males and females (Davoli et al. 2013), suggesting selection against mutations in ChrX may be less stringent. Population genetic studies of mCAs in participants of UKB have found that most mCAs have moderate to high fitness effects but occur at a low rate (Watson and Blundell 2023). Two notable exceptions are LOX and LOY, which are 1000‐fold higher in mutation rates than autosomal mCAs (Watson and Blundell 2023). In agreement, ChrX monosomy is the overwhelming cause of monosomy‐related early pregnancy loss (47/50), with autosomal monosomies being rare and limited to chromosomes 21 and 22 (Arnadottir et al. 2025). This raises the questions of how ChrX is lost and are there are unique characteristics of ChrX itself that make it loss‐prone?

Cell division is a major source of chromosome loss, because of the complexity of chromosome replication, cohesion, alignment, disjunction, and segregation (Guo et al. 2019). Cytogenetic analysis in 2048 cultured lymphocytes from a healthy 62‐year‐old woman has revealed that ChrX represents 1/4 of all lagging chromosomes, 6 times more often than would be expected by chance (1/23) (Catalán, Falck, and Norppa 2000). In line with this, the leading LOX‐associated germline variants are indeed enriched in genes regulating kinetochore and spindle function and chromosome segregation during mitosis, such as *TP53*, *MAD1L1*, *CENPQ*, and *CENPW* (Figure 3). It has long been proposed that premature centromere division (PCD)—the precocious loss of centromeric cohesion—is a mechanism of nondisjunction that causes ChrX monosomy and trisomy in blood cells and skin fibroblasts (Fitzgerald et al. 1975). There is a strong and significant correlation between the frequency of ChrX aneuploidy and PCD in lymphocytes of female patients with Alzheimer's disease (AD) and age‐matched healthy elderly females (Spremo‐Potparević et al. 2015). Several inherent variants in ChrX near the centromere are highly linked to LOX (Liu et al. 2024), further supporting that the ChrX centromere is involved in LOX mutation.

Formation of micronuclei, or micronucleation, is a biomarker of chromosome missegregation and an intermediate process to mediate chromosome loss (Guo et al. 2019) and rearrangement (Guo, Dai, et al. 2021). Important insights into the possible role of micronucleation in LOX mutation came from the cytogenetic investigation of ex vivo human lymphocytes. There is a significant increase with age in number of ChrX‐positive micronuclei in female lymphocytes than male lymphocytes (Hando et al. 1994; Richard et al. 1994; Wojda et al. 2007) and Xi is preferentially engulfed in micronuclei (Tucker et al. 1996). Moreover, there is a tenfold difference in the frequency of ChrX‐positive micronuclei in 46,XX females compared to 46,XY males and 45,X females, supporting the micronucleated ChrX is the Xi (Hando et al. 1997). However, several studies have shown that Xa and Xi are almost equally missegregated (Catalán, Falck, and Norppa 2000) or micronucleated in female lymphocytes (Surrallés et al. 1996; Wojda et al. 2007). Whether Xa micronucleation occurs as an active mechanism or is merely an accidental event remains to be elucidated. The population genetic study predicted that rate of Xa loss is more than 70‐fold higher than rate of Xi loss (1.55 × 10^−6^ vs. 1.11 × 10^−8^ per year) (Watson and Blundell 2023), supporting that Xa is substantially affected by LOX, at least at the mutation stage. Collectively, these findings fuel the hypothesis that mitotic missegregation and subsequent micronucleation of ChrX is a mutation mechanism of LOX (Figure 4B). Considering micronuclei also drive complex chromosome rearrangement (Guo, Dai, et al. 2021), segmental ChrX‐mCAs events may also result from ChrX micronucleation.

What, therefore, might account for the preferential loss of Xi in women? In addressing this question, the unique features of Xi that change with age should be emphasized. Xi has a much‐increased propensity for mitotic errors than Xa. Compared to Xa, Xi is preferentially prone to PCD and the following nondisjunction (Abruzzo et al. 1985), probably because the interaction between Xist and cohesin subunits actively repels cohesin complexes from the Xi (Minajigi et al. 2015). While the rate of ChrX PCD has been known to increase with age (Fitzgerald et al. 1975), Xi seems to experience a more substantial age‐related increase of PCD because human Xi has recently been found to undergo age‐dependent reactivation, with an enrichment of reactivated genes related to sister chromatid cohesion (Rocca et al. 2026). In addition, compared with Xa, Xi might degenerate more rapidly and is more susceptible to genetic instability with age, for example, induced by a high mutation rate (Jäger et al. 2013), telomere shortening (Surrallés et al. 1999), replication asynchrony (Koren and McCarroll 2014) or accumulation of epigenetic variation (Liu et al. 2023). Interestingly, Xi replication occurs late and is faster than either Xa or autosomes (Taylor 1960; Poonperm et al. 2023), and late replicating is presumably linked to a higher load of point mutations in Xi (Stamatoyannopoulos et al. 2009; Koren and McCarroll 2014). While the expression of LINE1 (long interspersed element‐1) retrotransposon facilitates XCI at different levels (Chow et al. 2010), Xi accumulates a disproportionately higher mutational load, driven by preferential *de novo* LINE‐1 insertions originating from the Xa and autosomes (de los Rios Barreda et al. 2026). Moreover, Xi has long been known to locate at the nuclear periphery at interphase of the cell cycle (Pageau et al. 2007), probably because recruitment to the nuclear lamina by XIST RNA is required for ChrX‐wide silencing (Chen et al. 2016; Grigoryan et al. 2021). Because there is a direct link between nuclear chromosome positions, segregation error rates and micronucleus content (Klaasen et al. 2022), nuclear peripheral Xi is therefore more likely to be missegregated and micronucleated than Xa, which is commonly located in central nuclear regions. Additionally, although Xa and Xi respond with a similar sensitivity to X‐rays‐induced DNA damage, chromosomal aberrations in Xi are significantly higher than those in Xa after DNA repair is inhibited, suggesting DNA repair is less efficient in Xi than Xa (Surrallés and Natarajan 1998). This inequality might also be interpreted as the preferential localization of Xi towards the nuclear periphery, where damaged DNA usually fails to be rapidly sensed and repaired (Lemaître et al. 2014). Thus, high susceptibility to instability explains why Xi is preferentially affected by LOX (Figure 4B). In contrast to Xi, the structural and (epi)genetic stability of Xa is rigorously safeguarded by master factors, such as the deacetylase SIRT7 (Simonet et al. 2026).

Emerging evidence supports that loss of Xi is likely adriven by a different mechanism involving the unusual 3D conformation of Xi. A unique structural feature that distinguishes the Xi from all other chromosomes is that Xi is partitioned into two extremely large intervals, called superdormains, separated by a boundary that contains the microsatellite repeat *DXZ4* (Bonora et al. 2018). Another unique structural feature of Xi is the presence of superloops, large chromatin loops that are often anchored at *DXZ4* (Darrow et al. 2016). To reach across several megabases to form superloops, DXZ4 needs to interact with two other ChrX‐linked microsatellite repeats *FIRRE* and *ICCE*, which also loop to each other (Bansal et al. 2020). These three microsatellite repeats are highly polymorphic in copy number of each repeat unit, which form long‐range intrachromosomal interactions mediated by CTCF and its cofactor YY1 (Jégu et al. 2017; Bonora et al. 2018). Recently, in a seminal study (Si et al. 2026), copy number variations of *ICCE* and *DXZ4* are found to strongly associate with LOX levels across UKB and All of Us. Females with longer arrays of *ICCE* or *DXZ4* are associated with increased LOX in *cis*, but not in *trans*, suggesting a direct effect of longer *ICCE* or *DXZ4* repeats on increased missegregation. The effects of *ICCE* and *DXZ4* are additive, contributing to a 4‐fold range in the likelihoods of different ChrX to be lost. Moreover, two variants within the *DXZ4* array appearing to reduce CTCF and YY1 binding disrupt the effect of *DXZ4* on LOX, suggesting that increased CTCF and YY1 binding at longer *DXZ4* arrays reduces segregation fidelity of the Xi. Thus, while the hub of *ICCE* and *DXZ4* is dispensable for the establishment of XCI (Jégu et al. 2017), they are essential for mitotic stability of Xi. However, it remains unknown how alterations in Xi packing drive Xi instability. Moreover, a recent study found that, akin to Xi, human hepatic Xa also possesses a bipartite superdormain conformation, despite the boundary locations being different between Xi and hepatic Xa (Xie et al. 2026). The bipartite structures of Xa emerged during hepatocellular carcinoma development. These findings raise the question of whether hepatic Xa conformation change is associated with its subsequent loss predisposition.

In sum, we propose the intricate three‐way interplay among genome‐wide germline variants, environmental stresses and factors lie within ChrX itself could drive the LOX mutation (Figure 4A). Upon XCI, Xa and Xi adopt distinct chromosomal architectures, localize to different subnuclear positions, and mutate at varied rates and scales. These features, acting either independently or synergistically, contribute to distinct predispositions of somatic loss between Xa and Xi.

### 3.3 Battle of Clones: ChrX‐Linked Competitions in LOX

Addressing the expansion rate of mCAs is difficult as it requires serially sampling blood specimens over longer time periods in large cohorts. Recently, a longitudinal study in participants of the PLCO cohort reveals that the average population expansion rate of LOX is 0.3% per year age (Kelly et al. 2026). The most straightforward working model to account for age‐related expansion of LOX cells is that reduced expression of ChrX genes provides selective advantages to LOX cells. In this sense, seeking the single or multiple driver genes in ChrX whose copy number loss will provide a selective growth advantage or disadvantage (we refer to these genes as “LOX genes”) is of great importance (see below). In addition, the mixture of LOX and wildtype non‐LOX cells (mosaic LOX) in blood inevitably entails interactions between them, which may determine the fate of LOX cells in a non‐cell‐autonomous manner. Cells compete for space and survival with neighboring cells in tissues throughout the lifespan that can be subdivided into three general models (Figure 5A) (van Neerven and Vermeulen 2023). The high cell turnover renders the hematopoietic system particularly prone to dynamic selection and cell competition (Waldvogel et al. 2024). Meanwhile, in cases of mosaic forms of aneuploidy, the interaction of aneuploid and euploid cells creates an ideal condition for in vivo cell competition (Fusari and Milán 2026).

**FIGURE 5:**
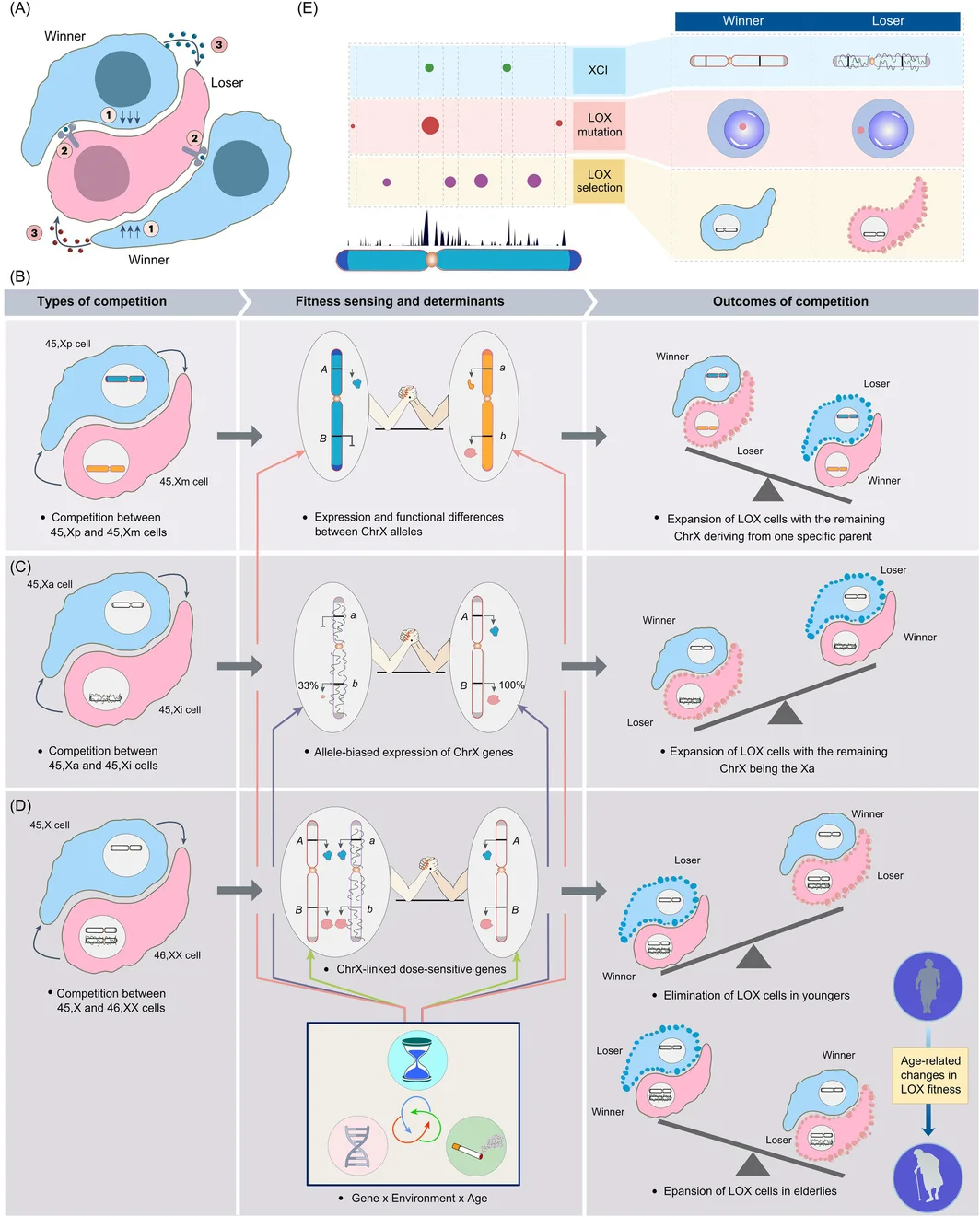
ChrX‐linked competitions (XLC) in loss of ChrX (LOX). (A) General models of cell competition. Interaction between two groups of cells with different fitness leads to cell competition. Under the framework of cell competition, the viable but less fit cells (losers) will be sensed and eliminated by neighboring fitter cells (winners), by the direct mechanical cues (①), cell–cell contact dependent signaling cues (②), or cell contact independent secreted factors (③) (van Neerven and Vermeulen 2023). (B) XLC between 45,Xp and 45,Xm cells. When LOX preferentially affects Xi in individuals with random XCI, competition will be set up between 45,Xa cells expressing either Xp or Xm. Xp and Xm differ by at least ~150 non‐synonymous coding variants and thousands of non‐coding variants (Boone et al. 2025). Importantly, 59 missense coding variants exhibit significant allelic shifts in LOX cases (Liu et al. 2024). In addition, ChrX‐linked imprinting preferentially expresses one of the Xp and Xm alleles. These effects aggregately lead to expression and function differences between Xp and Xm alleles, thereby resulting in fitness differences between 45,Xp and 45,Xm cells. The outcome is that 45,X cells carrying high‐fitness ChrX variants are preferentially expanded. (C) XLC between 45,Xa and 45,Xi cells. When LOX equally affect Xi or Xa, competition will be set up between 45,X cells retaining either Xa and Xi. In such a scenario, the fitness difference between 45,Xa and 45,Xi cells is determined by allele‐biased expression of ChrX genes. For example, 77% of genes are constitutively silenced in Xi, and the expression from Xi at escape genes reaches levels on average at 33% of expression from Xa (Tukiainen et al. 2017). The outcome is that 45,Xi cells are generally outcompeted by 45,Xa cells. (D) XLC between 45,X and 46,XX cells. In such a scenario, the fitness difference between 45,X and 46,XX cells is determined by ChrX‐linked dose‐sensitive genes, which are generally expressed higher in 46,XX cells compared to 45,X cells. The competition outcomes are age dependent, leading the 45,X cells to be eliminated in youngers but to expand in elderlies. The outcome of each competition scenario illustrated in (B–D) is determined by a set of specific factors (such as germline variants, environments, and age) that may be varied across females, thereby resulting in inter‐individual heterogeneity in the landscape of LOX. Overall, clonal expansion of LOX could be linked to Darwin's principle of natural selection, whereby the best adapted 45,X cells outcompete their neighbors (46,XX cells or less adapted 45,X cells) and populate with their own progeny. (E) Cause and consequence of XLC related to LOX. Dozens of variants spanning ChrX have been found to associate with LOX and the identity of retained ChrX, with varied effect sizes (Liu et al. 2024; Si et al. 2026). Depending on the potential function of these variants, their influences on LOX could be divided into three major categories: impacting ChrX inactivation choice, LOX mutation, and LOX selection. For example, variants within or around the Xp11.1 and Xq23 may influence the choice of which ChrX will be silenced. Such competition occurs before LOX, and the losers will be silenced and preferentially impacted by LOX. Variants around the centromere and telomere may influence the predisposition of mitotic missegregation. Such competition occurs during LOX, and the losers will be micronucleated. Other variants may influence the selection of LOX cells by altering functions of ChrX‐encoded proteins. Such competition occurs after LOX, and the losers will be eliminated.

Xp and Xm from each XX female differ by at least ~150 non‐synonymous coding variants and thousands of non‐coding variants (Boone et al. 2025). Intra‐individual diversity in clones expressing either Xp or Xm that are different in sequence content can trigger competitive interactions between clones, an emerging concept known as ChrX‐linked competition (XLC) (Boone et al. 2025). Buenaventura et al. (2024) elegantly studied a form of XLC by focusing on ChrX‐linked coding variation in the human *STAG2* gene. They found that competition between Xm‐expressing and Xp‐expressing clones is a non‐cell‐autonomous mechanism modulating the contribution of ChrX‐linked genetic diversity to organismal development in female mice (Buenaventura et al. 2024). Moreover, XLC has been observed in a mouse model of disorders related to *CASK*, a ChrX gene regulated by XCI. The survival rate of neurons is lower in female mice that have a mixture of *CASK‐*wildtype cells and *CASK*‐knockdown cells than in mice that only have *CASK*‐knockdown cells (Mori et al. 2025).

Considering the heterogeneity in LOX patterns, LOX individuals have a mixture of at least six cell types differing in dosage, activation status, or parental‐of‐origin in ChrX (Figure 2D). Adjacent cells with distinct LOX patterns may consequently express distinct ChrX gene repertoires. Considering differential fitness across clones is an essential element to set up competition (van Neerven and Vermeulen 2023), at least three mutually non‐exclusive competition scenarios exist in LOX individuals. We coin the term “XLC after LOX” to describe these specific competitions.

In first scenario, competition occurs between two subclones of 45,X with the Xa retained—45,Xp and 45,Xm. As two copies of ChrX alleles do not function in the same cells, function divergence between two alleles triggers the competition between 45,Xp and 45,Xm clones (Figure 5B). A possible inequivalent function between 45,Xp and 45,Xm has gained support from TS patients. As described above, 75%–90% of TS patients retain the Xm (Jacobs et al. 1997). One additional explanation for this inequality is the preferential miscarriage of 45,Xp embryos (Grande et al. 2019; Arnadottir et al. 2025), reflecting Xp is less efficient than Xm in enabling the survival of ChrX monosomic embryos. Generally, LOX cells expressing mutant alleles (such as 45,Xp) that confer proliferation advantages will outcompete LOX cells expressing normal alleles (such as 45,Xm). In support of this, evidence has shown that 44 independent ChrX‐linked alleles have shifted allelic fractions on the retained ChrX and 59 coding variants in ChrX exhibit significant allelic shifts in LOX individuals, suggesting they may confer differentiate selective advantages between 45,Xm or 45,Xp cells and result in clonal dominance of 45,Xm or 45,Xp cells in LOX individuals (Liu et al. 2024).

The second scenario is competition between 45,Xa and 45,Xi cells (Figure 5C). Most genes on Xi are transcriptionally silenced, and expression of escapees remains on average at 33% of Xa expression (Tukiainen et al. 2017). Therefore, loss of Xi might be less harmful than loss of Xa and subsequent selection may be less stringent against loss of Xi. In other words, 45,Xi cells under the general conditions are the less fit ones that are generally outcompeted by the fitter 45,Xa cells. This view is corroborated by three lines of evidence. Firstly, early cytogenetic studies have shown that, even if LOX mutation equally affects Xa and Xi (Surrallés et al. 1996), subsequent selection seems to preferentially remove 45,Xi clones (Abruzzo et al. 1985). Secondly, the simulation study has revealed that loss of Xi could be the event that drives 2‐fold higher fitness effect as compared to loss of Xa, with the growth rates being 15.7% and 7.3% per year, respectively (Watson and Blundell 2023). Given the critical role of Xp11.1 and Xp23 in choosing which ChrX to be inactivated (Rao et al. 2014), the fact that 45,Xa cells confer a selective advantage over 45,Xi cells may explain why two common variants on Xp11.1 (near *DXZ1*) and Xq23 (near *DXZ4*) can weakly increase the risk of LOX while strongly influencing which ChrX is lost in expanded clone (Loh et al. 2018). Thirdly, recent data revealed that most of the LOX‐related germline variants on ChrX are centered at or near the escapees and they tend to associate more strongly with LOX in *trans* than in *cis* (Si et al. 2026). Of note, the detection of 45,Xi cells in a small portion of LOX individuals (Machiela et al. 2016) indicates 45,Xi cells may be selected for stochastic reasons that require further study.

The third scenario is clonal competition between 45,X and 46,XX cells (Figure 5D), a prerequisite for successful clonal expansion of LOX (Figure 4C). Pathway‐based analyses suggest that mechanisms related to DNA damage repair, cell cycle regulation and apoptosis are critical for the clonal expansion of LOX (Liu et al. 2024). While LOX reduces cell anchorage independent growth in p53 deficient RPE1 cells under un‐challenged conditions (Chunduri et al. 2021), two lines of evidence have shown that LOX cells are positively selected within human body under certain contexts. Firstly, the increase of LOX prevalence in aging population suggests that competition outcome between 45,X and 46,XX cells is age dependent, probably because fitness effect of LOX itself changes over time or selection pressure on ChrX ploidy becomes much less stringent during aging. However, it will be necessary to carefully explore active forces that play a role in selection for and against 45,X cells. Secondly, exposure to cigarette smoke is associated with expanded LOX rather than detectable LOX (Liu et al. 2024), indicating that smoking might not only directly induce LOX, but also promote the expansion of pre‐existing LOX clones. However, this effect seems to be cohort‐dependent, because smoking does not significantly impact the expansion of LOX in PLCO (Kelly et al. 2026). These data highlights that competition between 45,X and 46,XX cells may be impacted by environmental stressors. How LOX cells obtain proliferative advantage under stressed conditions? One plausible mechanism is that, as LOX preferentially affect the late‐replicating Xi, 45,Xa cells could have a reduced S period and, as a consequence, reduced length of cell cycle, thereby providing a proliferative advantage relative to 46,XX cells (Levan and Mitelman 1979). Although this hypothesis has been proposed for nearly half a century, it has not been rigorously tested yet. Another alternative mechanism is that, as discussed in detail below, LOX cells obtain proliferative advantage and robust competitiveness by working in concert with other genomic instability events.

Together, the within‐individual presence of heterogenous clones with differential fitness fosters XLC after LOX, which could be influenced further by germline variants, age, and environment. An additional factor that can shape cell competition and selection in the immune system is antigens at the cell surface (Adams et al. 2020). In this regard, LOX results in two groups of 45,X immune cells with different ChrX‐linked surface antigens, leading to cell competition during clonal selection. In agreement with this, three germline variants near the *HLA* alleles (Figure 3A) influence the risk of LOX, probably through selecting ChrX‐linked cell surface antigens (Liu et al. 2024). Considering the high number of ChrX variants between Xp and Xm (Boone et al. 2025), XLC after LOX may be widespread.

In total, the mutation‐selection diagram (Figure 4A–C) predicts that LOX pattern is shaped by pre‐mutation biases and post‐mutation selection. Under this framework, more attention should focus on dozens of variants emanating from ChrX that are preferentially retained in LOX carriers (Liu et al. 2024). We speculate that these variants probably have *cis* or *trans* effects on XLC before, during, and after LOX (Figure 5E). For example, variants on Xp11.1 and Xq23 may determine which ChrX to be silenced (XLC before LOX); variants near ChrX centromere, such as rs6612886 and rs4029980, may predispose a specific ChrX to be missegregated (XLC during LOX); and variants mapped in the coding region of ChrX genes may influence the fitness of LOX cells retaining this specific ChrX (XLC after LOX).

## 4 Less ChrX, More Problems: Clinical Outcomes Associated With LOX

Clinical association of LOX remains an active area of research. Remarkably, a large‐scale epidemiological study with deep genotypic and detailed clinical data from four national‐scale biobanks has evaluated disease outcomes associated with detectable or expanded LOX and has shown that the phenotypes associated with LOX are diverse and exhibit high inter‐individual and inter‐biobank variabilities (Liu et al. 2024).

Among the most notable diseases associated with LOX are hematological cancer and autoimmunity. Association between LOX and acute myeloid leukemia (AML) can data back to 1979 (Levan and Mitelman 1979). In UKB, females with blood LOX have increased risk of lymphoid malignancies (Lin et al. 2021; Brown et al. 2023), but not myeloid malignancy (Brown et al. 2023). Recent evidence indicates that these effects are bigger than previously assumed. Liu et al. (2024) examined the association of LOX with 1253 diseases across 8 biobanks and found that detectable LOX is separately associated with risk of leukemia overall, AML and chronic lymphoid leukemia that is increased by 1.7‐, 1.9‐ and 3.3‐fold respectively. Clone size of LOX determines the strength of diseases association: Expanded LOX (CF > 5%) was associated with an even greater risk of these outcomes (6.3 folds for leukemia overall; 10.6 folds for AML; 14.7 folds for chronic lymphoid leukemia). In addition, significant associations with expanded LOX and overall leukemia risk in participants of UKB remained when individuals with other CH events were removed (3.8 folds after removing autosomal mCAs; 6.2 s fold after removing CHIP; 4.5 folds after removing autosomal mCAs and CHIP), indicating LOX is independently associated with leukemia risk (Liu et al. 2024). In this sense, LOX could be regarded as a premalignant mutation. These findings emphasize the importance of sufficient statistical power for detecting robust association between LOX and hematological malignancies. In addition to peripheral blood cells, when LOX presents as a major clone in bone marrow, it is often associated with hematopoietic diseases like AML, myelodysplastic syndrome and chronic myelomonocytic leukemia; when LOX represents a minor clone, it is likely to be an aging effect (Tang et al. 2016). Therefore, LOX provides the opportunity to better understand the molecular etiology of leukemogenesis. Of note, a recent study in two large Danish cohorts with 184,053 females finds that the risk of hematological cancer is not increased for individuals carrying LOX (Maeng et al. 2026). A drawback of this study is that effect of expanded LOX in hematological cancer was not analyzed. Besides leukemia, LOX is significantly higher in peripheral blood of females patients with severe aplastic anemia, a syndrome of markedly declined production of marrow blood cells (Strauss et al. 2024).

Autoimmunity is a set of more than 90 diseases driven by the aberrant attacking of solid tissues by immune cells (Libert et al. 2010). Autoimmunity predominantly affects women and several aspects of ChrX and its physiology, such as genes escaping XCI, skewing of XCI, and the presence of extra ChrX, underpin such bias (Libert et al. 2010). Cytogenetic studies have shown that LOX is higher in peripheral blood cells (particularly T and B lymphocytes) of female patients with primary biliary cholangitis (previously known as primary biliary cirrhosis) (Invernizzi et al. 2004; Miozzo et al. 2007), systemic sclerosis and autoimmune thyroid disease (Invernizzi et al. 2005), and other autoimmune diseases (Invernizzi et al. 2004) excluding systemic lupus erythematosus (Invernizzi et al. 2007) than healthy controls. A note of caution is that these studies usually involved a small number of patients. At present, some of these results have been replicated in large‐scale cohorts. For instance, expanded LOX (CF > 5%), but not detectable LOX, is significantly associated with heightened risk of systemic sclerosis and its subtypes in Japanese (Nishio et al. 2026). Moreover, recent studies revealed a more complex relationship between LOX and autoimmunity. A cross‐biobank study has shown that expanded LOX (CF > 10% in Biobank Japan and > 5% in UKB) confers an about 3‐fold increased risk of Graves' disease and rheumatoid arthritis, particularly in elderlies (≥ 65 year‐old) (Sonehara et al. 2025). However, in another Japanese‐based cohort study, LOX is consistently associated with reduced risk of young‐onset or late‐onset rheumatoid arthritis (Uchiyama et al. 2025). These data argue for a non‐linear relationship between LOX and rheumatoid arthritis.

LOX also associates with disorders outside the hematopoietic system. Cross‐biobank data has shown that LOX is associated with an increased risk of bacterial pneumonia (Liu et al. 2024). In addition, LOX was associated with a 3.9‐fold increase in the risk of arterial embolism and thrombosis in the Million Veteran Program (Liu et al. 2024). In 27,001 LOX females in FinnGen, individuals carrying a higher ChrX differential score (an indicator of higher LOX) were significantly associated with an increased risk of multiple subtypes of and any death from cardiovascular diseases (Liu et al. 2024). In populations without serious cardiovascular disease at baseline in UKB, detectable LOX in postmenopausal women is inversely associated with atrial fibrillation risk but is no longer significant after correction for multiple testing (Lim et al. 2024). In a large retrospective study of 773 suicides in Japan, LOX is significantly increased in suicides compared to controls (Otsuka et al. 2025). Besides, LOX females in UKB have an increased risk of acute tonsilitis (Lin et al. 2021). While LOX is not associated with the risk of spontaneous abortion in females (Nowinski et al. 1990; Russell et al. 2006), LOX women with a CF ≥ 0.87% have a 2.16‐fold lower likelihood of natural conception, and such association is independent of conventional ovarian reserve markers (Kikuchi et al. 2026), suggesting non‐expanded LOX is also potentially pathogenic.

While LOX is frequently observed in solid tumors (Box 3), it is surprising to see that blood LOX is not strongly associated with increased risk of solid cancers. Liu et al. (2024) found that detectable LOX is moderately associated with increased risk of skin cancer and benign neoplasm of the eye. Moreover, they found that expanded LOX is associated with increased risk of cancers in urinary organs (including the kidney and bladder). However, these associations are limited by low statistical power.

BOX 3 **LOX in tumors.**

LOX is exceptionally widespread in tumor cells. Using SNP genotyping to measure the number of autosomes and sex chromosomes in 620 cancer cell lines, Xu et al. found that, over time, female cells tend to lose Xi (Xu et al. 2017). Loss of Xi has been observed in a cancer cell line derived from a patient of Klinefelter syndrome (Kawakami et al. 2004). To address whether the in vitro systems faithfully recapitulate human in vivo tumors, Qi et al. (2023) presented a comprehensive pan‐cancer analysis of LOX across > 5000 tumors from The Cancer Genome Atlas data. In their analysis, LOX was defined as ≥ 99% of the ChrX that has been lost. They found that 14% of female tumors (757/5394) harbored LOX, pointing to the fact that LOX is a prevalent but understudied oncogenic alteration in cancer. Different tumor types show wide variation in the percentage of LOX. Within individual cancer types, the frequency of LOX can range from 0% (thymoma) and 1% (thyroid cancer) to 43% (uveal melanoma) and 56% (chromophobe kidney cancer) (Qi et al. 2023). Reasons for these differences remain elusive, but may be due to the cell type‐dependent rate of LOX mutation and fitness advantage offered by LOX. LOX in tumors is accompanied by nearly full loss of *XIST* expression and a 26% decrease of PARs gene expression (Qi et al. 2023). Besides full LOX, partial LOX, defined as at least one segment on ChrX being lost (Figure 1C), has been occasionally detected in female cancers (Qi et al. 2023). Partial LOX is associated with partial loss of *XIST* expression, probably depending on whether the long arm (where *XIST* locates) is lost (Qi et al. 2023). Considering the significant correlation between LOX and loss of *XIST* expression, an intriguing question is whether loss of *XIST* expression is an indicator of Xi loss, or perhaps a reflection of Xi reactivation. Notably, loss of Xa has also been indicated in several female cases of bladder cancer (Cheng et al. 2004).

LOX could be a driver event in some female cancers. For example, LOX cases of veal melanoma had increased rates of metastasis and this effect is not driven by increased overall chromosomal instability in LOX tumors (Qi et al. 2023), suggesting LOX enables some tumors to escape the immune system. Given that LOX is frequently observed in immune cells and solid tumors, it raises several fundamental questions, such as whether LOX in immune cells and solid tumors are inter‐connected, whether LOX immune cells interplay with LOX tumor cells, and if so, how LOX immune cells determine the clinical outcomes for patients with LOX tumors. Addressing these questions may build on existing knowledge and lessons from research on LOY (Abdel‐Hafiz et al. 2023; Chen et al. 2025). Interestingly, human ChrX is unusually enriched for tumor suppressor genes relative to autosomes (Davoli et al. 2013), partially explaining the observation that frequent LOX is observed in metastatic cancers. Observing LOX at a higher frequency in metastatic cancer suggests LOX is a driver event, but experimental studies are needed to provide the proof.

LOX has a context‐dependent relation to clinical outcomes of patients. In meningiomas, a women‐biased intracranial tumor, LOX is detected in about 10% of the cases and is associated with a significantly worse progression‐free survival (Berghaus et al. 2025). In contrast, while LOX is frequently detected in female AML patients with reciprocal translocation t(8;21) (Levan and Mitelman 1979), some results have shown LOX is associated with better outcomes in AML (Chen et al. 2020). The cancer‐type‐specific effect thus adds a layer of complexity to understanding the clinical significance of LOX in female tumors.

A critical question is how tumor cells respond to LOX. This question has been addressed only in cell lines. Following LOX, many tumor cells duplicate their retained ChrX, resulting in a state with two identical Xa (ChrX isodisomy, a state of copy‐number neutral loss of heterozygosity throughout the entire ChrX) (Xu et al. 2017). Maintenance of two Xa provides selective advantages for cancer cells. While TS‐derived ChrX isodisomic iPSCs are subject to XCI (Luo et al. 2015), neither of the two Xa in cancer cells undergoes XCI (Xu et al. 2017). Moreover, Xi reactivation is another mechanism driving the conversion of XaXi cells to XaXa in human breast and ovarian cancers (Pageau et al. 2007; Chaligné et al. 2015). In female embryonic stem cells, carrying two Xa results in reduced homologous recombination efficiency, lowered DNA repair activity, and increased genomic instability (Tamura et al. 2021). While these events are lethal for normal cells, they may provide fitness advantages for cancer cells.

In general, associations between ChrX‐mCAs and clinical phenotypes are typically weak and heterogeneous. Among 67,390 females from 19 National Heart, Lung, and Blood Institute Trans‐Omics for Precision Medicine program studies, there is no association between ChrX‐mCAs and lymphoid or myeloid cancers (Jakubek et al. 2023). In peripheral blood leukocytes of sub‐Saharan Africa children, ChrX‐mCAs are more likely to be detected in girls with Burkitt lymphoma compared to girls without Burkitt lymphoma (Zhou et al. 2023). Cross‐biobank data have shown that, while ChrX‐mCAs are not associated with hematologic cancer, they are marginally associated with increased lung cancer risk (Machiela et al. 2016). By contrast, ChrX‐mCAs are not associated with lung cancer after adjusting for age‐associated increase based on data from an international lung cancer cohort (Cheng et al. 2023).

Reconciling these results, LOX seems to be a predisposing factor to increased risk of hematological cancers and several benign diseases including autoimmunity, pneumonia, and suicide, with larger LOX clones conferring higher risk (Figure 1F). Of note, associations between LOX and disease risk vary across different cohorts, suggesting that the clinical consequence of LOX is influenced by demographic factors and genetic ancestries. Given that these results emerged relatively recently and ongoing studies enable a great range of diseases to be analyzed in diverse populations, the spectrum of LOX‐associated clinical manifestations is still expanding. Considering LOX expands exclusively in females, it might represent a novel driver of sex difference in many age‐related diseases (Bronikowski et al. 2022). In addressing how LOX might contribute to disease, a phenotypic understanding of LOX at all biological levels is needed. In the next sections, we attempt to integrate what is known about ChrX biology to enable current knowledge gaps in LOX to be dissected at molecular, cellular and organismal levels.

## 5 Molecular and Cellular Impacts of LOX

The clinical significance of LOX raises a critical question of whether LOX plays a causal role in the development of these diseases or is a byproduct of disease development. Understanding this question is not without challenges due to the scarcity of experimental models (Box 4). Despite this, we provide a summary of functional consequences of LOX (Figure 4D). Notably, insights from aneuploidy research, especially those from TS (Box 5), have greatly refined our current understanding of LOX. We posit that biological impacts of LOX could be caused by the cumulative effects of ChrX‐monosomy specific response and the general response to chromosome monosomy.

BOX 4 **Uses and challenges in modeling LOX.**

While human cohort‐based studies have uncovered the phenotypes associated with LOX, reliable in vivo and in vitro experimental models that mimic human‐like complexities are needed to inform new mechanisms underlying the cause and consequence of LOX.

Animal models of LOX allow organism‐level investigations. Encouragingly, mouse models of hematopoietic LOY have been built up (Sano et al. 2022; Zhang et al. 2022), providing a general framework to parallelly construct LOX mouse via CRISPR/Cas9‐mediated targeted ChrX elimination (Zuo et al. 2017). One important advantage of modeling LOX in mouse is that ChrX is the only chromosome to retain large‐scale synteny between mouse and human (Chinwalla et al. 2002). Another advantage is that there are subtle differences in behavior between 39,Xp and 39,Xm female mice, suggesting ChrX‐linked imprinting in mice does influence cognition and emotion in ways that are reminiscent of TS subjects (Davies et al. 2005; Skuse 2005). These comparable phenotypes suggest that molecular and mechanistic insights gained from mouse models are valuable for our understanding of human LOX. However, they failed to faithfully recapitulate key human physiology. For instance, mice display a higher tolerance to ChrX monosomy than humans, as 99% of human 45,X conceptuses are prenatally lethal, but ChrX monosomic mice (39,X) are viable and anatomically normal (Ashworth et al. 1991), probably because only 3%–7% ChrX genes escape XCI in mice (Berletch et al. 2015), compared with at least 23% in humans (Tukiainen et al. 2017). Most laboratory mouse strains also fail to capture menopause (Gilmer et al. 2023), a key phenotype that impacts the pathological association of LOX (Lim et al. 2024). In addition, age‐associated CH observed in humans is not shared by female laboratory mice, partly because of infrequent HSPCs self‐renewing divisions (Kapadia et al. 2025). Although whether spontaneous LOX occurring in the mouse hematopoietic system remains ripe to study, different mutation turnover rates in time between humans and laboratory mice should be considered.

Moreover, an in vitro human experimental system is necessary to complement traditional animal studies. ChrX monosomy cells can be created by CRISPR/cas9 or derived from spontaneous ChrX missegregation in mitosis (Chunduri et al. 2021). As LOX occurs autonomously during reprogramming of sex chromosome hyperploid cells (Box 1), human iPSCs lines that are autosomally isogenic but carry different sex chromosome complements can be obtained. Isogenic cells uniquely enable us to study LOX in context with minimizing confounders like genetic variability. An emerging cellular approach for studying LOX involves the generation of isogenic iPSCs and subsequent differentiation to various cell types. For example, random LOX during the reprogramming of 47,XXX cells gives rise to isogenic 46,XX and 45,X iPSCs. These iPSCs can be differentiated into a set of cell types for transcriptional and functional analyses to elucidate how LOX contributes to immunity dysfunction and disease pathology. Modeling mosaic LOX could be achieved by mixing 46,XX cells with 45,X cells, and models of increasing complexity could be built up by introducing equimolar concentrations of sex hormones to further our understanding of interaction between LOX and sex hormones. More importantly, sex hormone concentrations in culture can be dynamically adjusted to model the onset of menopause or pregnancy (Kouthouridis et al. 2022). In isogenic models, LOX‐related genetic variants could be introduced via gene editing to refine variants affecting LOX acquisition or variants influencing clonal expansion. Isogenic 45,Xp and 45,Xm cells differentiated from iPSCs can be used to study XLC before, during, or after LOX and its cell‐type specific effects. Similarly, this model can be used to discern environmental stressors, as well as their interaction with genetic variants, that could drive the acquisition of LOX mutation or drive the selective expansion of already formed LOX clones.

In sum, each model of LOX is limited by failing to fully capture human‐like complexities, which should be carefully considered if we want model‐based findings to be more robust and translationally relevant. To overcome inherent limitations of in vivo and in vitro models, the pathological role of LOX can be studied in vivo by transplanting human isogenic cells into mice models. For instance, in a preprint work, Davis et al. recently transplanted microglia derived from human isogenic iPSCs carrying 46,XX and 45,X karyotypes into *App* ^*NL‐G‐F*^ mice, and found that LOX impairs a broad range of immune and metabolic pathways implicated in microglial response to amyloid‐β pathology in AD (Davis et al. 2025). Other approaches like the advanced microphysiological models, which refer to small‐scale 3D models of human tissues like organoids and organ‐on‐a‐chip systems (Kouthouridis et al. 2022), are also applicable. These platforms, combined with computational modeling and multi‐omic analyses, are exciting emerging approaches to study LOX at the molecular and cellular levels.

BOX 5 **Functional consequences of ChrX monosomy: lessons from TS.**

Studies directly examining TS subjects have begun to shed light on the functional architecture of LOX. In most cases, 45,X could be catastrophic for embryonic development, as recent evidence has shown that ChrX monosomy is the most commonly embryonic lethal aneuploidy (9.9%) (Arnadottir et al. 2025; Wang et al. 2026) and up to 99% of fetuses with ChrX monosomy are believed to end in spontaneous abortions early in pregnancy (Gravholt et al. 2023). Impairment of development by ChrX monosomy is illustrated in TS children who survive infancy, who have significantly increased risk for solid cancers, autoimmune diseases, and cardiovascular diseases (Cameron‐Pimblett et al. 2017; Gravholt et al. 2023). In addition, TS is associated with an increased risk of psychiatric disorders (Gravholt et al. 2023) and neurodevelopmental disorders including autism spectrum disorder (Berry et al. 2024). The genetic etiology of TS is becoming unraveled. Haploinsufficiency for genes located in ChrX PARs (Figure 1E) is a primary predisposing factor for defects observed in TS (Gravholt et al. 2023). As an example, *SHOX* is the common gene located in ChrX PAR1 and its haploinsufficiency has been convincingly implicated in the short stature in TS (Rao et al. 1997). Another example is that haploinsufficiency of *CSF2RA*, a gene located in ChrX PAR1, could account for high embryonic lethality in 45,X conceptions (Urbach and Benvenisty 2009). While the heightened autoimmunity in TS females has long been attributed to the haploinsufficiency for immune‐modulatory genes from PARs and ChrX gametologs (Libert et al. 2010), a potential novel mechanism may in part be a less complex T cell receptor repertoire due to presentation of antigens from ChrX monosomy by thymic epithelial cells (Gylemo et al. 2024).

Considering the paradox that ChrX monosomy is the only monosomy compatible with life and the enrichment of highly dosage‐sensitive genes in human ChrX, it is believed that all viable 45,X cases are cryptic mosaics with some degree of undiscovered mosaicism (Fernández‐García et al. 2000; Hook and Warburton 2014). About 15% to over 50% of TS subjects carry the most common mosaic karyotype—45,X/46,XY mosaicism (Fernández‐García et al. 2000; Gravholt et al. 2023; Wang et al. 2024). The 45,X/46,XY mosaic TS subjects could help to understand age‐related LOX in the general population, because they are karyotypically similar to LOX females. One difference between them is that ChrX monosomy is presented in higher grades of mosaicism (measured by CF) in mosaic TS subjects than this in LOX females. For example, while the median CF of LOX is approximately 2%–4% in general women (Liu et al. 2024; Maeng et al. 2026), the estimated CF of 45,X cells per sample is 15%–79% among 186 UKB women of mosaic TS (Tuke et al. 2019). An urgent question therefore is what criteria should be used to distinguish TS and LOX individuals based solely on blood LOX data.

There is a general principle that mosaic aneuploidy is less serious than constitutional aneuploidy (Biesecker and Spinner 2013). On one hand, ChrX monosomy mosaicism attenuates the phenotypic severity of TS subjects. For example, the phenotypes (except the renal disease and comorbidities) in children with TS may increase with the proportion of mosaicism (Wang et al. 2024). Presence of 45,X cell clones in blood does not adversely affect reproductive lifespan or fertility in most women, as long as more than 20% of cells are 46,XX (Tuke et al. 2019). Neuroanatomy and cognitive performance in TS subjects can significantly predict the degree of 45,X mosaicism (Karipidis et al. 2026). Because of mild phenotypic impacts, some of 45,X/46,XX mosaics were never diagnosed (Tuke et al. 2019). On the other hand, the clone size of LOX (inferred via CF) determines the strength of its association with leukemia, infectious diseases, and immune diseases (Tang et al. 2016; Liu et al. 2024; Sonehara et al. 2025). Thus, different levels of mosaicism may partially explain why clinical associations between TS and LOX populations are largely unshared (Figure 1F). For instance, while pneumonia risk is heightened in TS and LOX populations (Schoemaker et al. 2008; Liu et al. 2024), suicide risk is increased in LOX (Otsuka et al. 2025) but not in TS (Thunström et al. 2024).

Overall, studies in TS subjects have provided important mechanistic insights into the functional consequence of LOX. Phenotype heterogeneity in 45,X/46,XX mosaics informs us that a correlation between LOX clone size and phenotype severity should be determined. Mouse models with controlled induction of LOX mutation could be used to address whether the clone sizes of LOX correlate with the developmental stage of the mutation induction and to determine the threshold of LOX clone size for pathogenicity of a given disease. The similarities between LOX and 45,X/46,XX mosaics suggest that results of LOX and TS studies are mutually complementary.

### 5.1 LOX Results in ChrX Haploinsufficiency

Some genes are categorized as haploinsufficient, as deleting a single copy of one allele in diploids leads to phenotype alterations or decreased organismal fitness (Chunduri et al. 2022). There is an enrichment of highly dosage‐sensitive genes in mammalian ChrX (Pessia et al. 2012). Given that LOX entails deletion of the entire ChrX and, hence, decreased expression of most ChrX genes *in cis*, it is easy to envision that such extensive insufficiency of ChrX genes could underpin some of the functional consequences of LOX (Figure 4D‐i).

About 3%–7% of ChrX genes are able to avoid XCI from onset during development (constitutive escapees) (Loda et al. 2022). For example, PARs genes and gametologs are present in two copies in both sexes and most of the ChrX gametologs are not inactivated in Xi (Figure 1E) (Loda et al. 2022). Because these genes are dosage‐sensitive, their biallelic expression from both ChrX is biologically important for women. On one hand, the gradually acquired ChrX haploinsufficiency may be one factor contributing to the etiology of LOX‐associated diseases. In keeping with this, it has been proposed that gradually acquired haploinsufficiency for specific ChrX‐linked immune‐modulatory genes in peripheral T and B lymphocytes contributes to the generation of autoantibodies, disturbances in self‐recognition and ultimately to autoimmunity (Invernizzi et al. 2005; Libert et al. 2010). In addition, LOX is associated with impaired ribosomal biogenesis and protein translation, which could be attributed to the haploinsufficiency of *RPS4X* (Chunduri et al. 2021), an XCI escaping gametolog that has been implicated in the etiology of TS (Fisher et al. 1990). On the other hand, haploinsufficiency of ChrX‐linked dose‐sensitive genes seems to be a determinant of competition between 45,X and 46,XX cells (Figure 5D), because sensing the altered expression of dose‐sensitive genes drives the selective elimination of aneuploid cells under physiological conditions (Fusari and Milán 2026). An emerging ChrX‐linked cell competition gene is *RPS4X*, because data from *Drosophila* has shown that differences in ribosomal protein gene dose between cells triggers cell competition to eliminate aneuploid somatic cells (Ji et al. 2021).

Evolution of ChrX dosage compensation illustrates that the mammalian genome is highly adaptative. To survive ChrX haploinsufficiency, mammalian stem cells could upregulate the remaining Xa through XCU. XCU is induced by ChrX monosomy to compensate for the dosage imbalance of ChrX genes with those on diploid autosomes (Talon et al. 2021; Allsop et al. 2025). This suggests that the loss of one ChrX can be sensed and compensated via increased transcription of the retained ChrX. The upregulated genes are enriched for dosage‐sensitive genes and are involved in protein complexes and important cellular functions (Allsop et al. 2025). XCU operates on a gene‐by‐gene basis at RNA and protein levels (Rücklé et al. 2023; Allsop et al. 2025). XCU may explain, at least partially, why ChrX monosomy is viable in humans and mice (Ashworth et al. 1991). However, phenotypic defects in TS subjects, as well as heightened pathogenic predisposition of LOX individuals, emphasize that XCU is not sufficient for completely rescuing ChrX monosomy. An immediate question that arises is whether XCU efficiency becomes weakened upon human aging.

### 5.2 LOX Sacrifices the Resilient Factors in Xi

Since Xi has historically been referred to as “inactive”, the biomedical significance of Xi has barely been explored. Advances in investigative techniques and model systems have driven a recent wave of research interest in Xi. As ~23% of genes on Xi are not subject to XCI, Xi has been thought to be important contributors for sexual dimorphism by acting as a female‐specific buffering mechanism (Khramtsova et al. 2023). For example, while hemizygous males are highly susceptible to recessive mutations on ChrX, females who carry a recessively mutant allele would not be affected if a functional allele is carried on the second ChrX. Female resilience is carried out by two buffering mechanisms (Migeon 2006). Firstly, a functional allele can be expressed in half of cells because the mutant allele is silenced at random. Secondly, even in the remaining 50% of cells where the mutant allele is expressed, the deleterious allele is sufficiently buffered by the functional allele if it has escaped XCI. Thus, Xi is considered a female protective factor against autism and other male‐biased congenital and developmental disorders (Talukdar and Page 2026). Supporting this theory, a recent study used human induced pluripotent stem cells (iPSCs)‐derived brain organoids to demonstrate that alleles on Xi serve as a critical reservoir dynamically used along neural differentiation, thereby ameliorating neurodevelopmental disease phenotypes in females (Bertin et al. 2026). As a comparison, ChrY contributes directly to males' heightened susceptibility to autism spectrum disorder (Berry et al. 2024).

Besides acting as a buffer mechanism, several persuasive lines of experimental evidence from cells without Xi or with more Xi have demonstrated that Xi is functionally active. Our initial knowledge about the functions of Xi comes from the subtraction of features observed in normal subjects from those seen in subjects with sex chromosome aneuploidies. Sex differences in inflammatory response activation and resolution in tumor‐associated macrophages enriched for microglia are facilitated by genes expressed or modulated by Xi, demonstrating a pivotal role for Xi in protecting against glioblastoma multiforme incidence and mortality in females (Tharp et al. 2025). The *trans* role of Xi in modifying autosomal gene expression was confirmed by another study, in which the majority (95%) of differentially expressed genes are autosomal in 45,X human iPSCs derived from 46,XX fibroblasts (Meleckyte et al. 2025). Using human cells with varied sex chromosome number, David Page and colleagues provided the most direct evidence that Xi has a remarkably similar effect in modulating the expression of genes on autosomes (San Roman et al. 2024) and Xa (San Roman et al. 2023), in both in vitro cell lines and in vivo primary cells (Blanton et al. 2024). Remarkably, among four cell types of hematopoietic lineage, Xi can elicit cell‐type‐specific responses on modulating the expression of autosomes and Xa (Blanton et al. 2024). In agreement with data from cell lines, whole genome transcriptomic analysis validated that 45,X fetal samples (11–18 weeks post conception) completely lacking Xi exhibit dysregulated expression of several autosomal genes with regulatory functions as compared with matched 46,XX control samples (Suntharalingham et al. 2025). *ZFX*, an XCI escaping gametolog, represents a major gene acting *in trans* to modulate autosome and Xa (San Roman et al. 2024). The discovery that Xi regulates autosomal genes fits well with results from high‐throughput chromosome topology studies that have shown that few escapees are engaged in long‐range interactions with each other and with the autosomal active compartment (Splinter et al. 2011).

Outside of its baseline activity, mammalian Xi tends to become partially re‐activated upon aging (Hoelzl et al. 2025; Rocca et al. 2026). ChrX reactivation could be driven by several age‐related factors, such as telomere shortening (Schoeftner et al. 2009), accumulation of DNA methylation variability in Xi (Liu et al. 2023) or reduction of LaminA/C‐defined repressive nuclear periphery (Grigoryan et al. 2021). After ChrX reactivation, biallelic expression of specific ChrX genes may confer advantage or disadvantage in different contexts. On one hand, Xi in female lymphatic tissues and lymphocytes show a stronger degree of escape (Tomofuji et al. 2024) and are more predisposed to become partially reactivated (Wang et al. 2016). This leads the ChrX‐linked immunity‐related genes to be overexpressed in females, thereby increasing their susceptibility for autoimmunity (Wang et al. 2016). On the other hand, studies in mouse models have shown that Xi is able to counter age‐related cognitive decline (Davis et al. 2020), via baseline (Shaw et al. 2023) and age‐induced transcriptional escape in genes critical to learning and memory (Gadek et al. 2025). In this regard, Xi is a female‐specific factor that may confer female resilience in cognitive decline in aging. Considering this, LOX may increase the risk of dementia. In support of this, a cytogenetic study based on multiprobe quantitative FISH have demonstrated that the aggregate rate of LOX and ChrX gain is significantly increased in cells of hippocampus and prefrontal cortex in patients with AD (Yurov et al. 2014). In addition, reduction in ChrX read depth in female dorsolateral prefrontal cortex is associated with reduced cognition and faster rate of cognitive decline (Graham et al. 2019). Although so far there are no epidemiological studies indicating that LOX in blood is associated with dementia risk, we cannot exclude this possibility (see below).

Although XIST has long been perceived as a *cis*‐acting RNA, that is, XIST is expressed from and binds exclusively to Xi (Figure 1E), recent studies have expanded XIST's role beyond XCI. In mouse models, loss of Xist not only leads to Xi reactivation, but also induces a cascade of genome‐wide dysregulation of genes involved in DNA replication, chromosome segregation, cell‐cycle checkpoints, and hematopoiesis, thereby promoting a highly aggressive myeloproliferative neoplasm and myelodysplastic syndrome that eventually progress into myelofibrosis and leukemia (Yildirim et al. 2013). The underlying molecular mechanisms are not fully understood, but several pathways have emerged. In both human and mouse cells, XIST/Xist RNA is able to spread to spatially close autosomal sites and coat specific autosomal regions, where it regulates chromatin structure and genome‐wide gene expression (Dror et al. 2024; Yao et al. 2025). In addition, XIST RNA has a multifaceted role in genome regulation beyond XCI, such as function as a transcriptional attenuator and microRNA sponge (Morey et al. 2025).

Altogether, it is conceivable that loss of the potential resilient factors and genome‐wide regulators in or expressed from Xi may contribute to the etiology of LOX‐associated diseases (Figure 4D‐ii), pointing that special attention on Xi is merited. Although we highlight the protective role of Xi to females, it is important to note that, for an equivalent reason, alterations in Xi also pose increased disease risk to females (Cáceres et al. 2025).

### 5.3 LOX Results in Skewed Usage of Parental ChrX

Cellular consequences of LOX may largely depend on the activity of the remaining ChrX. As mentioned above, XLC after LOX typically results in skewed retention of parental ChrX, raising the questions of whether skewed usage of parental ChrX has consequences with respect to the clinical phenotypes of LOX carriers, and if so, how?

In addition to minor differences in DNA sequence, two ChrX in females exhibit parental‐specific DNA methylation, named ChrX‐linked imprinting, that preferentially expresses one of the parental alleles (Chen and Zhang 2020). ChrX‐linked imprinting in humans was firstly suggested by the observation that TS females of 45,Xp are significantly superior in social cognition to those of 45,Xm (Skuse et al. 1997). Likewise, there is a significant variability in visual memory and brain anatomy between 45,Xm and 45,Xp individuals (Bishop et al. 2000; Kesler et al. 2003; Jean‐Francois et al. 2013). Stronger evidence for different expression and function of Xm and Xp in females is coming from functional studies. As noted above, XCU is induced in female mouse stem cells with ChrX monosomy, regardless of which ChrX is remained (Talon et al. 2021). However, expression from the Xm‐linked allele tends to be slightly higher than that from the Xp‐linked allele (Allsop et al. 2025). In a mouse model, Xp has more methylation in CpG islands than Xm in autoantigen‐stimulated CD4^+^ T lymphocytes, suggesting Xp and Xm play different roles during the immune response (Golden et al. 2019). Using a transgenic female mouse model in which all cells are enforced to use Xm as the only Xa, Abdulai‐Saiku et al. (2025) found that, compared to mosaic XCI mice, these mice exhibit impaired cognition and accelerated biological and epigenetic aging in the hippocampus. Mechanically, CRISPR‐mediated activation of the Xm‐imprinted genes *Sash3*, *Tlr7*, and *Cysltr1* improved cognition in aging Xm female mice. Besides, an early study has reported that *Xlr3b* is an imprinted candidate for ChrX‐linked parent‐of‐origin effects on cognitive function in mice (Davies et al. 2005).

Based on evidence described above, skewed usage of parental ChrX after LOX has implications to our understanding of AD, the most prevalent dementia. Increased frequency of PCD and ChrX aneuploidy (including LOX) have been observed in lymphocytes and brain cells of female AD patients (Spremo‐Potparević et al. 2008; Yurov et al. 2014; Spremo‐Potparević et al. 2015). ChrX exerts a critical role in the human brain (Jiang et al. 2025) and a reduction in ChrX read depth (an indicator of LOX) in the female dorsolateral prefrontal cortex is associated with cognitive decline in a manner independent of age (Graham et al. 2019). Coupling these results with the finding that mammalian Xm is less functional than Xp in memory and cognition (Abdulai‐Saiku et al. 2025), LOX may provide potential novel explanations for the heterogeneity and sexual dimorphism in clinical characteristics of AD. Firstly, considering the functional difference between Xp and Xm, skewed representation of them when LOX preferentially affects ChrX specifically derived from one parent may lead to phenotypic variation among female patients of AD. For example, in LOX‐positive AD patients, where Xm is predominately remained in LOX cells, may have different clinical manifestations as compared to those with Xp being predominately remained. Secondly, considering ChrX in men is strictly the less‐functional Xm, it may provide novel insight to explain why males with AD progress faster and have a worse outcome than females (Davis et al. 2020).

The “loss of mosaicism” is a main hypothesis proposed to explain why XCI skewing is associated with autoimmunity (Stewart 1998; Libert et al. 2010). In light from skewed usage of parental ChrX in LOX cases, this hypothesis can be adapted to explain why LOX is associated with heightened risk of autoimmunity. As the human antigen‐recognition system (T cells in thymus and B cells in bone marrow) works mainly to detect and eliminate offending pathogens, one critical capacity of this system is to avoid eliciting destructive reactivity to self‐constituents. To establish self‐tolerance, developing T and B cells interact with a myriad of ‘self’ molecules (positive selection) and those with high avidity for self‐antigens are culled with high fidelity (negative selection). In general females, random XCI results in two populations of dendritic cells that express either Xp‐ or Xm‐linked self‐antigens for thymic negative selection of T cells. Conversely, if a woman has biased LOX that, for example, preferentially results in loss of Xp, T cells will be largely tolerized by dendritic cells expressing Xm‐linked self‐antigens. Hence, a small proportion of autoreactive T cells specific for Xp‐linked self‐antigens will escape negative selection and be exported to the periphery. Then, these non‐tolerized Xp‐reactive T cells will activate B cells presenting the same endogenous Xp‐linked self‐antigens. If this model holds true, varied degrees and distinct patterns of LOX across females can contribute to inter‐individual heterogeneity of autoimmunity risk.

Together, intra‐individual variation in ChrX affected by LOX results in a mosaic population of 45,X cells unequally retaining Xp or Xm (Figure 4D‐iii). Differences in epigenetic imprinting and genetic polymorphism between Xp or Xm lead to expression heterogeneity in ChrX genes, thereby contributing to important biological and phenotypic variations among LOX cells. Future clinical association studies of LOX will be more informative if parental‐of‐ChrX retention in LOX individuals can be distinguished and analyzed precisely. One of the key challenges is that the parental DNA of most, if not all, biobank participants is lacking. Another challenge is that we need to precisely distinguish the parental‐of‐ChrX retention, especially when CF of LOX is low. Notably, Hofmeister et al. developed a parent‐of‐origin allele inference method for biobank‐scale datasets, without requiring parental genomes (Hofmeister et al. 2022; Hofmeister et al. 2025). This method enables high‐throughput inference of the parental origin of the retained ChrX in LOX individuals at an unprecedented scale.

### 5.4 LOX Is Associated With Increased Genomic Instability

In females, participants with ChrX‐mCAs are more likely to harbor autosomal mCAs (Machiela et al. 2016; Cheng et al. 2023), a more heterogeneous group of detectable Mb‐scale mCAs on chromosomes 1–22 (Dai and Guo 2021). Likewise, females with higher CF of autosomal mCAs are more likely to have LOX (Brown et al. 2023). In women of UKB, the most common autosomal mCAs‐LOX co‐occurrences are Chr22‐LOX and Chr13‐LOX, and the most frequent CHIP‐LOX co‐occurrences are *DNMT3A*‐LOX and *TET2*‐LOX (Barnao et al. 2026). However, germline variants associated with LOX and autosomal mCAs are largely unshared (Liu et al. 2024), suggesting they do not have shared germline predisposition. Interestingly, LOX carriers in UKB have increased risks of lymphoid and myeloid malignancies only when also harboring autosomal mCAs, and hazard ratio is further increased by co‐occurrence of CHIP (Brown et al. 2023). These observations are consistent with that CHIP and mCAs co‐occurrences are strongly selected for and associated with pronounced elevations in leukemia risk (Barnao et al. 2026). However, such co‐occurrence between CH types makes it inherently challenging to disentangle the pathological role of LOX per se.

To explain such co‐occurrence in LOX carriers, three mutually non‐exclusive mechanisms are plausible. Firstly, LOX leads to further genomic instability. This theory is supported by the observation that the level of sporadic aneuploidy in chromosomes 8, 15, and 18 is twice as high in cultured lymphocytes isolated from TS subjects as in lymphocytes from controls (Reish et al. 2006). Further supporting this, the prevalence of autosomal copy number variants is significantly increased in TS subjects (Prakash et al. 2016). However, this conclusion warrants scrutiny, as empirical evidence has shown that chromosomal instability is not increased in human p53‐deficient RPE1 cells with engineered LOX (Chunduri et al. 2021), supporting the idea that ChrX monosomy per se does not induce chromosomal instability (Hintzen et al. 2022). Hence, data regarding whether LOX could induce genomic instability are controversial, and further studies to clarify the impact of LOX on genomic instability in HSPCs are necessary. Secondly, some co‐occurring CH subtypes may be selected to increase survivability and proliferation of LOX cells and *vice versa* during the clonal expansion of mutant cells. It can be expected that, by lowering expression of tens to hundreds of ChrX genes, LOX could lead to fitness defects. Indeed, LOX inhibits cell proliferation, either in human engineered LOX cells (Chunduri et al. 2021) or in TS‐derived iPSCs (Luo et al. 2015). In this setting, accumulation of other CH events may help to eliminate LOX‐induced proliferation barriers. In agreement, mouse embryonic stem cells with functional LOX accumulate more transcriptomic signatures of autosomal aneuploidy over time in culture than their isogenic XX counterparts (Stanton et al. 2026). However, longitudinal data from the PLCO cohort showed that LOX and autosomal mCAs have cooperative expansion, though this association does not reach statistical significance (Kelly et al. 2026). Thirdly, the co‐occurrence of LOX and other CH events was determined by methods used on bulk DNA (Box 2), which provides the full spectrum of CH events occurring in all cells. Hence, it is plausible that LOX and other CH events may not essentially co‐occur in the same cell. Considering that the immune system is a surveillance to eliminate aneuploid cells (Santaguida et al. 2017), we posit that immune cells with LOX may be less efficient at clearing cells carrying autosomal mCAs. This is an exciting research theme, given that LOX is associated with dysfunction of the immune system (see below).

The association of LOX with genomic instability has obvious implications. While TS subjects could help understand LOX females (Box 5), disorders associated with these two populations are largely unshared (Figure 1F). One prominent example is cancer. While hematopoietic LOX in the general population is associated with increased risk of leukemia overall but a normal risk of solid cancers (Liu et al. 2024), women with TS have a normal risk of leukemia overall but increased risk of solid cancers (Ji et al. 2016). These data suggest that LOX alone cannot result in leukemia. In agreement, LOX on one hand is always associated with autosomal alterations in AML patients. For example, LOX has been observed in female patients of leukemia with the acquired t(8;21) (a translocation between chromosomes 8 and 21) (Levan and Mitelman 1979; Mohamed and Dun 2015) or other autosomal alterations (Settin et al. 2006). On the other hand, the strength of association between expanded LOX and leukemia risk (overall or subtypes) is attenuated when individuals with autosomal mCAs, CHIP, or both mCAs and CHIP are removed (Liu et al. 2024), suggesting LOX may also work in concert with other types of CH to influence leukemia risk and progression. Moving forward, a thorough analysis of which co‐occurring CH events facilitate LOX clones to acquire malignant potential is crucial for advancing our understanding of LOX‐leukemia association. The same logic has been verified in TS subjects. While ChrX monosomy alone is not always sufficient to cause congenital heart defects, a common duplication in chromosome 12p13.31 interacts with ChrX monosomy to cause congenital heart defects in TS (Prakash et al. 2016). The need for a second hit in causing congenital heart defects in TS may explain why LOX alone is not significantly associated with increased risk of cardiovascular diseases in the general population (Liu et al. 2024).

Together, the clinical phenotype of LOX carriers is probably determined by numerous complex mechanisms, one of which is seemingly the second genetic hit from genomic instability (Figure 4D‐iv). In this respect, LOX appears to be an initial step along the long road to disease that facilitates a path to the next stage: whole‐genome instability, either large or small in scale. Although data from the FinnGen cohort indicate that the acquisition of LOX likely precedes the acquisition of autosomal mCAs because the CF of LOX is much higher than that of autosomal mCAs (Liu et al. 2024), it should be confirmed by further analyses in co‐occurring cases, though such analyses will be challenged by limited sample size.

### 5.5 Consequences of Aneuploidy: ChrX Monosomy and Beyond

Current available evidence discussed above supports that functional consequences of LOX could arise from altered expression of genes located on the lost or retained ChrX (the direct effect) and/or from disruption of genomic homeostasis due to ChrX monosomy (the indirect effect) (Figure 4D). In addition to these effects that are induced in response to LOX, some effects of LOX may result from a general cellular response to chromosome loss. Since loss of any single autosome is embryonically lethal in humans, ChrX monosomy gives us the unique opportunity to evaluate the general organismal consequences of chromosome monosomy. Despite this, results from in vitro cell models provided important insights into this issue.

It is traditionally assumed that cellular consequences upon monosomy differ, depending on which chromosome is affected. However, some of these consequences are shared across monosomies. Analysis of various cell lines with engineered monosomic karyotypes (monosomy 7, 10, 13, 18, 19p, and X) derived from human immortalized hTERT‐RPE1 cell line reveals that ChrX monosomy has unique and shared cellular phenotypes with other human monosomies (Chunduri et al. 2021). One consistent cellular phenotype of all monosomies analyzed is impaired ribosome biogenesis and translation (Chunduri et al. 2021). It should be noted that this phenotype is conserved in ChrX monosomy arising from LOY in human embryonic stem cells (Sarel‐Gallily et al. 2025). Impaired homeostasis in protein and other macromolecular complexes in turn affects the function of cells, tissues, and organs. Because ribosomal protein genes are spread across the genome, defects in ribosome quality control seem to be a shared feature of monosomies (Chunduri et al. 2021) and trisomies (Escalante et al. 2025). Therefore, some phenotypes previously interpreted as a direct consequence of ChrX monosomy (Fisher et al. 1990) could be a common response to aneuploidy. In this regard, perspectives from much‐studied aneuploidy (Fusari and Milán 2026) would facilitate our understanding of LOX.

Collectively, deregulation of cellular networks observed in LOX cells results from the cumulative effects of the ChrX‐monosomy specific response and the general response to chromosome monosomy. Further studies are needed to equally focus on both aspects to improve our understanding of molecular mechanisms behind phenotypes of LOX.

## 6 Organismal Impacts of LOX

LOX‐induced changes in molecular and cellular networks will affect the properties of tissues and organs, thereby influencing organismal phenotypes. In this section, we outline the current state of knowledge on the organismal impacts of LOX, with a specific focus on immune dysfunction and aging (Figure 4E).

### 6.1 LOX Is Associated With Immune Dysfunction

In physiological hematopoiesis, HSPCs contribute to the daily production of blood cells that are required for tissue oxygenation and immunity. Disruption of normal hematopoiesis causes impaired production of red blood cells, platelets, or immune cells. Pathway‐based analyses have shown that LOX is significantly associated with HSPCs, hematopoietic cell lineage, and differentiation gene sets, suggesting mechanisms related to HSPCs differentiation are critical for the development and clonal expansion of LOX (Brown et al. 2023). In UKB, there are enriched associations between LOX and blood count traits, such as higher lymphocyte count and lower neutrophil count (Figure 4E‐i) (Brown et al. 2023; Liu et al. 2024). Interestingly, most of the leading LOX‐related variants identified from allelic shift analyses (35/43) are associated with at least one alteration in blood cell phenotypes (Liu et al. 2024). Similar to LOX, ChrX‐mCAs are associated with decreased counts and percentages of neutrophils but increased counts and percentages of lymphocytes (Jakubek et al. 2023).

Altered blood cell traits in LOX individuals may associate with immune dysfunction, which is supported by observation that LOX is associated with increased risk of diseases accompanied by clinical manifestations of immune dysfunction, such as hematological malignancies and autoimmune diseases (Figure 1F). In addition, expanded LOX is associated with an increased COVID‐19 IgG antibody serostatus in UKB, although no significance is achieved (Sonehara et al. 2025). A recent proteomic study in UKB found a specific association of LOX with upregulated proteins involved in cytotoxic lymphocyte/natural killer response and downregulated proteins involved in B‐cell/cytokine‐associated signaling (Weyrich et al. 2026). While this evidence indicates that LOX introduces unique challenges in immune regulation, it is important to note that the association between LOX and immune dysfunction may be confounded by shared risk factors. For instance, several LOX‐associated loci also implicate genes related to cancer and immunity (Figure 3). Anyhow, immune dysfunction may explain why blood LOX is associated with heightened risk of diseases for which blood is not the disease‐related tissue.

These results suggest that LOX frequently alters the differentiation trajectory of HSPCs and results in skewed hematopoiesis and altered immune response, probably because XLC after LOX alters HSPCs' behavior in response to proliferation, differentiation, or cell death cues. A recent study (Shi et al. 2026) provided novel insights into our understanding of how hematopoiesis and immunity is influenced by LOX. Shi et al. found a novel chimeric RNA, UBA1‐CDK16, which forms via *cis*‐splicing between the adjacent genes *UBA1* and *CDK16* located in Xi. UBA1‐CDK16 is almost exclusively found in blood, and functional studies strongly suggest this chimeric RNA is a regulator of hematopoiesis, potentially inhibiting myeloid differentiation in females. Since LOX preferentially impacts Xi, the fact that UBA1‐CDK16 is solely produced from Xi indicates that diminished expression of UBA1‐CDK16 may partially explain why LOX individuals have biased hematopoiesis and dysfunctional immunity.

### 6.2 LOX Is Associated With Aging Hallmarks

Owing to its association with age and contribution to disease risk late in life, LOX is conventionally viewed as a natural consequence of aging. However, whether LOX is a cause or a consequence of biological aging remains unknown. Aging is associated with a rapid cell loss due to decreased rate of cell proliferation and increased rate of cell death and senescence (López‐Otín et al. 2013). It is plausible that acquisition of LOX represents an adaptive strategy by cells to bolster tissue resilience, thereby better withstanding intrinsic and extrinsic stressors that accompany aging. In this context, enhanced fitness and augmented proliferation capacity of LOX cells could provide beneficial effects by enabling rapid tissue regrowth. Thus, LOX is critical for blood cells and HSPCs to adapt to aging‐related stresses in females. One key question in this realm is how age‐related pathways shift LOX cells from dormancy to clonal expansion (Figure 5E). Understanding molecular signals driving clonal expansion of LOX during aging could reveal intervention targets that can mitigate LOX expansion and related disease risk. A candidate event that may foster the positive selection of LOX is inflammaging, an age‐related condition accompanied by low‐grade persistent level of pro‐inflammatory molecules (Dai and Guo 2021). This remains ripe for study.

Moreover, an intriguing scenario is that LOX would accelerate aging. The spread of genome mosaicism has been suggested to drive the aging process through the loss of tissue homeostasis (Dai and Guo 2021). Could LOX conceivably have a causal role in the manifestations of processes involved in aging? Two lines of evidence support that LOX conceivably has a causal role in the manifestation of processes involved in aging. Firstly, one‐directional Mendelian randomization did not suggest a causal relationship between telomere attrition and LOX (Brown et al. 2023); instead, telomere length reduction in LOX individuals may be a marker of past growth and clonal expansion of LOX HSPCs. As telomere erosion is a driver of aging (López‐Otín et al. 2013), reduced telomere length in LOX clones could lead to further genomic instability and the acquisition of additional aging hallmarks. Considering this, it is critical to conquer whether telomere length is progressively shortened with increased clone size of LOX in females. Secondly, preliminary results have shown that subjects with detectable LOX display a 1.82‐fold acceleration in DNA methylation‐based epigenetic clocks (Young et al. 2025), suggesting LOX is a driver of epigenetic aging. This is not unexpected, as TS subjects exhibit genome‐wide DNA hypomethylation (Trolle et al. 2016) and LOX in propagating female mouse iPSCs is associated with genome‐wide methylation gain (Pasque et al. 2018).

Together, it is tempting to assume that LOX is both the cause and consequence of aging, forming a self‐reinforcing loop (Figure 4E‐ii) that underlies the mechanisms whereby LOX increases the risk of or exacerbates chronic diseases. Evaluating the mechanisms of LOX in encoding aging phenotypes requires determining its effect on multiple aging hallmarks and specifically reverting them to demonstrate causality in suitable experimental models (Box 4). Moreover, the validity of causality warrants further testing in longitudinal studies in women's cohorts.

## 7 Important Knowledge Gaps and Ways Forward to Address Them

Compared to LOY, LOX is a larger scale mutation because ChrX, either Xa or Xi, is larger in size and harbors more biologically important genes and functional variants than ChrY. However, LOX is not as well‐studied as LOY, due in large part to that LOX is less prevalent and phenotypically less severe. LOX and LOY have been studied as separate entities because they occur in different sexes and involve distinct chromosomes. Given the intertwined evolutionary histories of human ChrX and ChrY (Figure 1E), it is reasonable to assume that LOX and LOY are inherently interconnected. Insights from much‐studied LOY, therefore, can inform less‐studied LOX. For example, data from mice models have revealed that deletion of *Kdm5d* or *Uty*, two ChrY gametologs, is able to phenocopy the pathogenic attributes of LOY in acute myeloid leukemia (Zhang et al. 2022) or heart failure (Horitani et al. 2024). Since the ChrX gametologs *KDM5C* and *UTX* are XCI escapees (Figure 1E), future studies investigating whether they are LOX genes would therefore be extremely valuable. Thus, research focusing on LOX is vital to health equity between sexes and a source of novel discovery. To move forward, several areas deserve attention.

Firstly, a well‐recognized limitation is that most of our knowledge on clinical outcomes associated with LOX has come from studies in Europeans. Conclusions drawn from Europeans may not successfully be extrapolated to other groups. Although there are non‐European groups in some biobanks, these are relatively small. Thus, it is urgent to establish large‐scale biobanks that are based on non‐Europeans, especially populations of Africans and Latin‐Americans, not only to allow for replication of associations, but also to allow equitable research of LOX in diverse ancestries and races. African females show a much higher number of intra‐individual ChrX‐linked coding variants and Latin‐American females are more likely heterozygotic for rare ChrX variants due to a complex history of admixture (Boone et al. 2025). This means that it may foster robust XLC related to LOX (Figure 5E) in Africans and Latin‐Americans. If so, one could speculate that the pattern and clinical phenotypes of LOX carriers from African and Latin‐American may be different from those from other regions. In addition, although current studies based on population‐based biobanks, where the individual is the sampling unit, have answered several fundamental questions about LOX, plans for a new wave of family‐based biobanks, where the family is the unit of sampling, are underway (Davies et al. 2024). Family‐based biobanks offer particular advantages for LOX studies when genetic data of parent‐offspring trios are collected. For example, when data of parental ChrX are available, one can determine parental‐of‐ChrX loss and retention and their different consequences in LOX individuals.

Secondly, LOX is estimated from data of blood DNA sequencing that is often done once, therefore it represents a cross‐sectional snapshot, lacking information on the evolution and temporal duration of LOX clones. Future longitudinal studies aiming to serially sample blood specimens over longer time periods in large cohorts is required to ameliorate this challenge. Determining the long‐term intra‐individual dynamics of LOX landscape across time and cell types can provide powerful insight into longitudinal dynamics of XLC after LOX. In addition, integrating longitudinal data with mathematical models not only helps to quantify the expansion kinetics of LOX clones, while also disentangling relative contributions of cell‐intrinsic and cell‐extrinsic factors in LOX expansion (Watson and Blundell 2023; Marzban et al. 2026). Another benefit from longitudinal studies is that a cause‐and‐consequence relationship between LOX and disease development could be estimated. As UKB and other large‐scale biobanks intend to collect detailed information on longitudinal (i.e., decades or more) changes (Allen et al. 2024), it will greatly facilitate these endeavors. A recent study in PLCO cohort provided an important view of the longitudinal dynamics of LOX with unprecedented resolution (Kelly et al. 2026). Although this study provided a breadth of data that is otherwise difficult to obtain, it is limited by short follow‐up periods (median 4.2 years) and small LOX carriers (213).

Thirdly, since LOX was determined in bulk blood DNA in most biobank studies, within‐individual variation in LOX across blood cell types remains largely unexplored. This is important because (i) there are many distinct cell types in blood (Figure 4E), each of them or even each sub‐population of the same cell type may play a different role in disease development; and (ii) there is a wide intra‐individual heterogeneity in LOX patterns (Figure 2D). Under the setting of the whole body, the rate of LOX expansion may be influenced by diverse factors across tissues and developmental stages. For example, while LOX prevalence in blood increases with age, age‐related LOX is not evident in the brain dorsolateral prefrontal cortex (Graham et al. 2019). The cell‐type‐ and tissue‐specific landscape of LOX begs deeper investigation (Box 1), which is challenged by sampling different tissues from the same individual. Besides, recent technological advances in single‐cell DNA sequencing would enable us to understand the full genetic heterogeneity of LOX at single‐cell resolution and to reconstruct phylogenetic trees that track the evolutionary history of LOX in human tissues (Shao et al. 2025). As distinct selective pressures for mutant clones may arise during aging or disease pathogenesis (Brunner et al. 2026), separating the distinct LOX patterns that arise from biological processes or disease pathogenesis through single‐cell technologies could gain deeper knowledge about the heterogeneity in mechanisms from which LOX can arise and expand. Likewise, as there is a clear positive association between fitness effects of autosomal mCAs and blood cancer risk (Watson and Blundell 2023), the fact that loss of Xa and loss of Xi have significant differences in fitness effects enforces us to ask whether this holds true for LOX. Thus, LOX studies in the future should ideally incorporate the knowledge of which one between Xa and Xi is lost in a single genome. In addition, single‐cell transcriptomics, epi‐transcriptomics, and proteomics are yet‐to‐follow focuses, which would allow more detailed analyses of expression and regulation heterogeneity of LOX cells at the RNA and protein levels, thereby yielding information about the sources of phenotypic heterogeneity across LOX carriers. Overall, understanding the cell‐type‐ and tissue‐specific landscape of LOX may offer insights into disease‐specific mechanisms of LOX.

Fourthly, while there is an increasing body of literature supporting the association between LOX and disease risk, this evidence remains largely correlative. What we do not know is whether LOX causally contributes to disease pathology and, if so, which ChrX genes are the “LOX genes”. Is it the resultant effect of losing many ChrX genes with small effect or primarily due to loss of one major ChrX gene? These questions can be systematically studied in cell culture and animal models (Box 4). Identifying specific LOX genes remains an immense challenge because ChrX encompasses hundreds of genes. Besides, Mendelian randomization provides a method to test the causal relationships between LOX and diseases based on the already generated genetic and phenotypic data. Compared to female LOX, there is a long history of research bias towards male LOY (Guo, Li, et al. 2021). While mechanistic views on how LOY contributes to disease development have considerably expanded (Guo and Dai 2025), mechanistic studies on LOX are likely to continue this trend. For example, Ahern et al. generated iPSCs from human LOX fibroblasts and found that the derived neural crest cells show disrupted expression of genes involved in cholesterol metabolism, neural crest cell signaling and translation, and mitochondrial function (Ahern et al. 2025). To dissect precise molecular mechanisms of LOX, one mindset learned from aneuploidy (Tang and Amon 2013) is that some mechanisms are rooted in the dosage change of single LOX genes and some of them are the cumulated effects of a large number of independent single LOX genes.

Fifthly, while over 56 germline variants (Figure 3A) have been found to influence LOX risk, which of them influence predisposition to LOX mutation or the fitness of LOX cells is not well understood. Longitudinal data in the PLCO cohort show that germline susceptibility to LOX is not associated with expansion rate (Kelly et al. 2026). Hence, increased emphasis should be given to functional dissection. Among the already‐identified variants, the rs78378222 that maps in the 3′‐untranslated region of *TP53* should be one prime candidate for functional studies (Figure 3B), because experimental data have shown that RPE1 cells with LOX can survive only when *TP53* was knocked out or knocked down (Chunduri et al. 2021). Considering p53 controls a lot of distinct biological processes (Kastenhuber and Lowe 2017), exactly how rs78378222‐induced changes in the function or regulation of *TP53* lead to increased risk of LOX need to be fully delineated and proved. The rs78378222, which has a frequency of 1.92% in the Icelandic population, changes the consensus AATAAA polyadenylation signal to AATACA (Stacey et al. 2011). On one hand, this substitution reduces the efficiency of mRNA 3′‐end formation by 89% (Sheets et al. 1990). Subsequently, reduced mRNA 3′‐end length can affect mRNA stability, translation efficiency, and location, which finally result in diminished production of protein and altered intracellular communications (Manning and Cooper 2017). On the other hand, this substitution in mRNA alters its binding with microRNAs, such as miRNA‐125b (Zhao et al. 2016) and miR‐545‐3p (Macedo et al. 2016), thereby increasing microRNA‐mediated repression. In agreement, biological samples from rs78378222 carriers have lowered p53 protein levels (Macedo et al. 2016). Experimental data have shown that rs78378222 downregulates both p53 mRNA and protein levels and reduces cellular apoptosis (Li et al. 2013). Thus, decreased p53 expression represents a key mechanism underlying the LOX predisposition conferred by rs78378222. In our opinion, there are four cellular routes by which rs78378222 would increase LOX predisposition (Figure 3C). Investigating these four aspects would provide a deeper understanding of how germline variants confer LOX cell fitness advantages under the constraint of ChrX‐monosomy‐induced fitness loss. Moreover, as p53 functions decline in the aging process (Feng et al. 2007), lessons from functional characterization of rs78378222 will provide important insights into why LOX increases substantially in older populations. Likewise, mechanisms underlying germline predisposition to LOY could also benefit from these studies because rs78378222 is also linked to increased LOY predisposition (Wright et al. 2017). Another candidate gene deserving more attention is *SP140L*, because its association with LOX has been robustly replicated (Loh et al. 2018; Jakubek et al. 2023; Liu et al. 2024).

Finally, both genes and environments undoubtedly play a critical role in the rise of LOX. However, there are still substantial gaps in our understanding of their interplays, including gene–gene, gene–environment, and environment–environment interactions. These gaps can be fulfilled by (i) developing new computational tools to perform integrative analysis of the available large‐scale epidemiologic and genomic datasets; (ii) integration of environmental risk factors into longitudinal studies in large‐scale biobanks with diverse populations; or through (iii) direct functional validation in appropriate experimental models (Box 4). Of note, whether sex hormones, an individual's endogenous environmental exposure, influence the expansion and consequence of LOX clones is still to be explored. While sex hormones have a far‐reaching effect that can even supersede the genetic inclinations of certain cell types (Kouthouridis et al. 2022), estrogen is recently found to influence class‐switched memory B cell frequency only in humans with 46,XX karyotype (Peckham et al. 2025), suggesting the immune modulation effect of sex hormones is influenced by cells' sex chromosomal background. Sex hormones fluctuate extensively before and after menopause, raising the question regarding whether menopause is a residual confounding factor influencing the clinical manifestation of LOX. Illuminating in this regard is that LOX inversely associates with atrial fibrillation risk among postmenopausal women rather than premenopausal women (Lim et al. 2024). Although LOX is not associated with blood concentration of sex hormones, expanded LOX is associated with significantly increased blood levels of sex hormone‐binding globulin (Liu et al. 2024). Estrogen has been suggested to alter CH dynamics by driving the predominance of *DNMT3A*‐mutant cells in the blood of female mice (Stomper et al. 2025). These results point to a particularly possible scenario that LOX may be influenced by circulating levels of sex hormones or interact with sex hormones to exacerbate or mitigate the expansion of LOX clones and the manifestation of LOX‐associated diseases. Surgical removal of the ovaries of the LOX mouse model (Box 4) could be used to study the contribution of gonadal hormones to LOX pathophysiology. In addition, it has been shown that both germline variants and acquired LOX contribute to immune dysregulation (Sonehara et al. 2025). Therefore, the interplay between germline variants and acquired LOX in shaping disease risk and progression is an emerging field.

## 8 Conclusions

It is widely recognized that bi‐allelic ChrX can be beneficial to females (Migeon 2006); however, evidence from LOX studies clearly suggests that this can also be disadvantageous because one ChrX tends to be lost upon aging. Age‐related LOX in blood cells, first noted over 60 years ago (Jacobs et al. 1963), has recently gained much renewed attention for its prevalence in aging women and its potential role in influencing women's health. By rigorously reviewing the existing data from key discoveries in the past six decades, we have deciphered potential mechanisms underlying the acquisition and expansion of LOX and its functional and organismal impacts, although some of these have not yet been deeply demonstrated. Based on these advances, we posit that the pathogenic effects of LOX may depend on which ChrX is lost, when LOX rises and expands, where LOX is predominantly affected, what degree of LOX mosaicism is acquired, and to a lesser extent, the co‐occurring subtypes of CH (Figure 4F). While we still have many questions than answers about the cause and consequence of LOX, we expect that, once we have sufficient data, LOX may eventually be proved to have some causal roles in women's aging and age‐related diseases.

As pointed in this Review, recent years have brought major advances in clinical associations of LOX, our understanding about the underlying mechanisms and biological consequences, however, is still in its nascent stage. Studies of LOX have traditionally trailed behind these of LOY and increased general awareness around LOX is required. Given their pathophysiological similarities, there are important parallels between LOX and much‐studied LOY. Therefore, research on LOX may build on existing knowledge and lessons learned from research on LOY (Guo et al. 2020; Bruhn‐Olszewska et al. 2025; Guo and Dai 2025; Abdel‐Hafiz et al. 2026; Zhang et al. 2026). An emerging body of literatures indicates that LOX‐associated phenotypes do not solely rise from lacking the second ChrX. To in‐deep understand the cause and consequence of LOX, it should go beyond simply studying LOX, towards to a broader context of ChrX biology—including XCI and XCU, XCI escaping and skewing, Xi reactivation, ChrX monosomy and TS syndrome, XLC and parent‐of‐origin effects of ChrX—and aneuploidy. Further studies on LOX may not only provide novel insights into fundamental principles of genome aging in women, but also help to determine the potential value of LOX as a biomarker for diagnostic purpose and risk stratification applications and whether modulating LOX burden can be exploited to prevent or mitigate disease in women (Guo, Li, et al. 2021). Given increased interests in comprehensive examination of mutational landscape across human body and increased efforts in tool and technological development (Coorens et al. 2025; Brunner et al. 2026), we anticipate that important breakthroughs at greater resolution will continue to revolutionize the field of LOX.

## Author Contributions

**Xihan Guo:** conceptualization; funding acquisition; investigation; visualization; writing – original draft; writing – review and editing. **Xueqin Dai:** conceptualization; funding acquisition; investigation; writing – original draft; writing – review and editing.

## Funding

This work was supported by the National Natural Science Foundation of China (32260148, 82302957, 32560159), the Xing Dian Plan “Youth Talent Program” of Yunnan Province, and the Yunnan Fundamental Research Projects (202501AT070101, 202401CF070054).

## Conflicts of Interest

The authors declare no conflicts of interest.

## Data Availability

Data sharing not applicable to this article as no datasets were generated or analysed during the current study.
